# Modelling phytoplankton-virus interactions: phytoplankton blooms and lytic virus transmission

**DOI:** 10.1007/s00285-024-02093-w

**Published:** 2024-05-02

**Authors:** Jimin Zhang, Yawen Yan, Junping Shi

**Affiliations:** 1 School of Mathematical Sciences, Heilongjiang University, Harbin, 150080 Heilongjiang People’s Republic of China; 2 Department of Mathematics, William & Mary, Williamsburg, VA 23187-8795 USA

**Keywords:** Reaction–diffusion model, Phytoplankton blooms, Lytic viruses, Basic ecological reproductive index, Basic reproduction number, 92D25, 35K57, 92B05

## Abstract

A dynamic reaction–diffusion model of four variables is proposed to describe the spread of lytic viruses among phytoplankton in a poorly mixed aquatic environment. The basic ecological reproductive index for phytoplankton invasion and the basic reproduction number for virus transmission are derived to characterize the phytoplankton growth and virus transmission dynamics. The theoretical and numerical results from the model show that the spread of lytic viruses effectively controls phytoplankton blooms. This validates the observations and experimental results of Emiliana huxleyi-lytic virus interactions. The studies also indicate that the lytic virus transmission cannot occur in a low-light or oligotrophic aquatic environment.

## Introduction

Phytoplankton are the world’s most important aquatic producers. They play an essential role in biogeochemical cycles and strongly influence the abundance and biodiversity of aquatic communities. Light and nutrients are two essential resources for phytoplankton growth (Huisman et al. 2006; Klausmeier and Litchman 2001; Wang et al. 2007; Yoshiyama et al. 2009; Zhang et al. 2021). Phytoplankton absorb light energy to synthesize carbon dioxide and water into organic matter and release oxygen (Chen et al. 2015; Davies and Wang 2021). At the same time, they ingest various nutrients from the surrounding environment to preserve normal physiological metabolism (Vasconcelos et al. 2016; Zhang et al. 2018, 2021). These processes are important for achieving energy conversion and elemental cycles in nature and maintaining the carbon-oxygen balance of the atmosphere.

Viruses in aquatic ecosystems are generally small particles. The components of viruses are mainly nucleic acids and protein coats (Fuhrman 1999). This means that viruses can trigger the biosynthesis of the viral genome and protein only when they are parasitic in the host cells (Beretta and Kuang 1998; Edwards and Steward 2018; Fuhrman 1999; Fuhrman et al. 2011). Viruses are extremely abundant and widely distributed over oceans, lakes, and rivers (Suttle 2005). It is shown that viruses are the major pathogens and important causes of mortality for most aquatic organisms (Demory et al. 2021; Fuhrman 1999). As a result, viruses directly affect the structure and stability of aquatic communities.

It is recognized that viruses infect a significant proportion of phytoplankton and it is a major cause of the loss of phytoplankton (Demory et al. 2021; Kuhlisch et al. 2021; Suttle et al. 1990). According to the mechanism of virus transmission among phytoplankton, lytic viruses are considered to be one of the most common viruses (Beretta and Kuang 1998; Edwards and Steward 2018; Fuhrman 1999). The process of the lytic virus infection can be divided into the following steps. First, the virus contacts and adsorbs on phytoplankton cells by random diffusion, and then injects its nucleic acid into the cells. Second, the virus takes over the synthesis machinery of phytoplankton cells and produces viral genome and protein biosynthesis, which is needed for the viral offspring. Third, after the new virus is assembled, the lytic process ends with the lysis of the phytoplankton cell membrane, and then the virus particles are released into aquatic environments. This infection process indicates that lytic viruses destroy phytoplankton cells and reduce the biomass of phytoplankton. In view of the interrelationship between phytoplankton and lytic viruses, it is of great interest to explore the spread of lytic viruses among phytoplankton.

In this study, we propose a mathematical model to describe the spread of lytic viruses among phytoplankton. Here the aquatic environment under consideration is a poorly mixed water column. This suggests that both phytoplankton and virus distributions are spatially heterogeneous (Huisman et al. 2006; Klausmeier and Litchman 2001). Light comes from the water surface and nutrients come from the bottom of the water column (Ryabov et al. 2010; Yoshiyama et al. 2009; Zhang et al. 2021). Phytoplankton growth requires light and nutrients. Lytic viruses move randomly with turbulence and use phytoplankton cells as hosts to replicate and reproduce, eventually releasing a large number of lytic viruses when the cells rupture (Demory et al. 2021; Edwards and Steward 2018; Fuhrman 1999; Kuhlisch et al. 2021). One principal objective of the present paper is to model and elucidate the mechanism of lytic virus transmission among phytoplankton in a heterogeneous environment.

Several mathematical models have been introduced to investigate lytic virus transmission (Béchette et al. 2013; Beretta and Kuang 1998; Demory et al. 2021; Edwards and Steward 2018; Fuhrman et al. 2011). In these existing studies, the basic assumptions are that both phytoplankton and viruses are spatially uniformly distributed. However, there is growing evidence that only shallow aquatic environments and epilimnion are well-mixed, while most aquatic ecosystems are poorly mixed. This means that phytoplankton generally exhibit strong spatial heterogeneity (Huisman et al. 2006; Klausmeier and Litchman 2001; Ryabov et al. 2010; Yoshiyama et al. 2009; Zhang et al. 2021). Our model consists of four dynamic reaction–diffusion equations. Its contribution is the ability to describe the spatially heterogeneous distribution of phytoplankton and viruses. This model also incorporates the effects of light and phytoplankton sinking relative to existing models of lytic viruses. Thus the reaction–diffusion model contains advection terms and a nonlocal structure. This increases the complexity of the model structure and the difficulty of model analysis. We will rigorously derive the basic ecological reproductive index for phytoplankton invasion and the basic reproduction numbers for lytic virus transmission by analyzing nonnegative steady states and some basic properties of solutions of the model.

Phytoplankton blooms are an important phenomenon in which phytoplankton biomass increases rapidly and significantly over a period of time (Chen et al. 2015; Kuhlisch et al. 2021). It has adverse effects on aquatic ecological environments. Phytoplankton blooms lead to poor water quality of aquatic resources, cause the death of aquatic organisms, and even threaten the health and safety of humans (Ho et al. 2019). It has become apparent that lytic viruses can influence phytoplankton biomass abundance from observations and some experiments (Demory et al. 2021; Edwards and Steward 2018; Fuhrman et al. 2011; Kuhlisch et al. 2021; Suttle et al. 1990). For example, *Emiliana huxleyi* is distributed worldwide and frequently forms large and dense blooms. These blooms are often terminated by the lytic virus transmission (Kuhlisch et al. 2021). Experiments have shown that about 50% of *Emiliana huxleyi* cells are infected by a large double-stranded DNA virus during blooms, and 25–100% *Emiliana huxleyi* deaths are related to the lytic virus infection (Fuhrman 1999; Kuhlisch et al. 2021). Another objective of this study is to examine these observations and experimental results theoretically and reveal the evolution trend in phytoplankton biomass and free lytic virus density with varying ecological factors based on the mathematical model described above.

The structure of the paper is organized as follows. In the next section, a mathematical model consisting of four reaction–diffusion equations is formulated to describe phytoplankton-virus interactions. By using the principal eigenvalue theory, bifurcation theory, and persistence theory, we analyze some basic properties of dynamic solutions and nonnegative steady states of the model in Sect. 3. Two important basic indices are derived. In Sect. 4, numerical bifurcation and time series diagrams are made to explore the role of lytic virus transmission for phytoplankton blooms, and the changes in phytoplankton biomass and free lytic virus density for varying environmental factors. An overview of the main conclusions and future research questions are presented in the last section.

## Model formulation

**Fig. 1:**
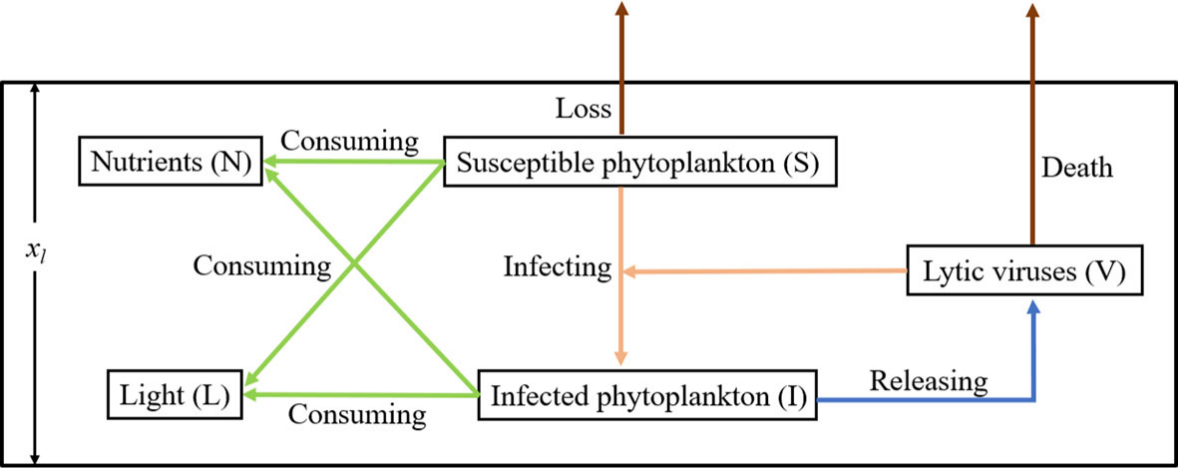
Phytoplankton-virus interactions in a poorly mixed water column

The water depth coordinate is *x* and the time scale is *t*. Consider a poorly mixed water column with $x=0$ at the water surface and $x=x_l$ at the bottom of the water column. The model consists of four reaction–diffusion equations describing the change of the concentrations of susceptible phytoplankton (*S*(*x*, *t*)), virus-infected phytoplankton (*I*(*x*, *t*)), free lytic virus particles (*V*(*x*, *t*)), and dissolved nutrients (*N*(*x*, *t*)). Their interaction relationship is shown in Fig. 1. The biological significance of variables and parameters in the model is summarized in Table 1.

**Table 1:** Variables and parameters with realistic values and biological significance of model (2.1)

Symbol	Meaning	Values	Units	Source
*t*	Time	Variables	day	
*x*	Depth	Variables	m	
*S*	Biomass density of susceptible phytoplankton	Variables	cells/m^3^	
*I*	Biomass density of virus-infected phytoplankton	Variables	cells/m^3^	
*V*	Density of free lytic viruses	Variables	virions/m^3^	
*N*	Concentration of dissolved nutrients	Variables	mgN/m^3^	
$d_p$	Vertical turbulent diffusivity of phytoplankton	1 (0.05–5)	m^2^/day	Grover (2017); Huisman et al. (2006); Jäger et al. (2010); Klausmeier and Litchman (2001); Ryabov et al. (2010); Yoshiyama et al. (2009)
$d_v$	Vertical turbulent diffusivity of free lytic virus particles	1 (0.05–5)	m^2^/day	Grover (2017); Huisman et al. (2006); Jäger et al. (2010); Klausmeier and Litchman (2001); Ryabov et al. (2010); Yoshiyama et al. (2009)
$d_n$	Vertical turbulent diffusivity of dissolved nutrients	1 (0.05–5)	m^2^/day	Grover (2017); Huisman et al. (2006); Jäger et al. (2010); Klausmeier and Litchman (2001); Ryabov et al. (2010); Yoshiyama et al. (2009)
$\omega$	Directional movement velocity of phytoplankton	0.1($-$0.2$-$0.6)	m/day	Grover (2017); Jäger and Diehl (2014); Jäger et al. (2010); Ryabov et al. (2010)
*r*	Maximum specific production rate of phytoplankton	1	day^-1^	Edwards and Steward (2018); Jäger and Diehl (2014); Wang et al. (2007)
$\mu _p$	Loss rate of phytoplankton	0.1	day^-1^	Jäger et al. (2010); Vasconcelos et al. (2016); Wang et al. (2007)
$L^0$	Light intensity at the water surface	300	$\mu$mol(photons)/(m^2^s)	Jäger et al. (2010); Wang et al. (2007)
$l_0$	Background light attenuation coefficient	0.4(0.1$-$2.5)	m^-1^	Jäger et al. (2010); Vasconcelos et al. (2016); Wang et al. (2007)
*l*	Light attenuation coefficient of phytoplankton	$6\times 10^{-10}$	m^2^/cell	Ryabov et al. (2010)
$\theta$	Proportion of virus-infected phytoplankton capable of photosynthesis and nutrient consumption	0.5(0–1)	–	Assumption
$\gamma$	Half-saturation constant for nutrient-limited production of phytoplankton	3	mgN/m^3^	Jäger and Diehl (2014); Vasconcelos et al. (2016)
*h*	Half-saturation constant for light-limited production of phytoplankton	100	$\mu$mol(photons)/(m^2^s)	Jäger and Diehl (2014)
$\beta$	Transmission coefficient between susceptible phytoplankton and lytic viruses	$3.6\times 10^{-3}$	m^3^cell^-1^day^-1^	Edwards and Steward (2018)
$\eta$	Intraspecific competition coefficient	0.002	m^3^cell^-1^day^-1^	Assumption
$\delta$	Lytic death rate (1/*T*)	1.2	day^-1^	Fuhrman et al. (2011)
$\mu _v$	Death rate of free lytic viruses	0.8(0.2$-$1.7)	day^-1^	Edwards and Steward (2018)
*q*	Lytic virus replication factor, known as “burst size"	10(2–30)	virions cell^-1^	Béchette et al. (2013); Edwards and Steward (2018); Fuhrman et al. (2011)
*b*	Infected cells per adsorbed virion	1	cells/virion	Edwards and Steward (2018)
$c_p$	Nutrient content per phytoplankton cell	0.01	mgN/cell	Assumption
$N^0$	Nutrient input from the bottom of the water column	50(0–500)	mgN/m^3^	Jäger and Diehl (2014); Vasconcelos et al. (2016); Wang et al. (2007)
$\alpha$	Nutrient exchange rate	0.05	m/day	Jäger and Diehl (2014); Vasconcelos et al. (2016); Yoshiyama et al. (2009)
$x_l$	Depth of the water column	10	m	Assumption

According to the research work in Beretta and Kuang (1998); Edwards and Steward (2018); Fuhrman et al. (2011), we have the following assumptions:

(A_1_): Only susceptible phytoplankton can reproduce through photosynthesis and consumption of nutrients;
(A_2_): Virus-infected phytoplankton are removed by lysis before reproducing. The latency period is *T* from the infection to the lysis. The lytic virus reproduces inside phytoplankton cells during this period *T*;
(A_3_): The lysis of virus-infected phytoplankton releases massive amounts of lytic virus particles.

Let *S*(*x*, *t*) and *I*(*x*, *t*) be the biomass density of susceptible phytoplankton and virus-infected phytoplankton, respectively. They have two different forms of movement in the water column. One is random movement in the vertical direction by turbulence with a diffusivity $d_p$ (Huisman et al. 2006; Klausmeier and Litchman 2001; Ryabov et al. 2010; Yoshiyama et al. 2009; Zhang et al. 2021). The other is directional movement including sinking or buoyant due to gravity or seeking more light with a velocity $\omega$ (Grover 2017; Klausmeier and Litchman 2001; Ryabov et al. 2010; Yoshiyama et al. 2009). The whole water column is a closed environment for phytoplankton. This means that *S*(*x*, *t*) and *I*(*x*, *t*) satisfy no-flux boundary conditions at endpoints $x=0$ and $x=x_l$.

Whether virus-infected phytoplankton can consume resources is still a disputed subject (Gourley and Kuang 2004). Here we introduce a parameter $\theta \in [0,1]$, which represents the proportion of infected phytoplankton capable of resource consumption (Smith and Thieme 2012). By Assumptions (A_1_) and (A_2_), the growth of susceptible phytoplankton depends on light and nutrients. It is expressed as

$$\begin{aligned} rg(N)f(L(x,S+\theta I))S=r\cdot \displaystyle \frac{N}{\gamma +N}\cdot \displaystyle \frac{L(x,S+\theta I)}{h+L(x,S+\theta I)}\cdot S. \end{aligned}$$

Here the light intensity $L(x,S+\theta I)$ following the Lambert-Beer law (Huisman et al. 2002) is given by

$$\begin{aligned} L(x,S+\theta I)=L^0\exp \left( -l_0x-l\displaystyle \int _0^x(S(z,t)+\theta I(z,t))dz\right) ,~x\in (0,x_l) \end{aligned}$$

since light is absorbed by water and phytoplankton above the point *x*. The reduction of susceptible phytoplankton biomass includes three parts: $-\mu _sS$ (death and grazing Jäger et al. 2010; Vasconcelos et al. 2016; Wang et al. 2007; Zhang et al. 2021), $-\eta (S+\theta I)S$ (competition among phytoplankton for other growth resources such as inorganic carbon for photosynthesis Davies and Wang 2021; Hsu et al. 2017; Nie et al. 2016; Zhang et al. 2021), and $-b\beta SV$ (lytic virus infection into virus-infected phytoplankton (Beretta and Kuang 1998; Edwards and Steward 2018; Fuhrman et al. 2011)). The increase in virus-infected phytoplankton biomass comes from $b\beta SV$ and the loss is owing to the lysis with the lytic death rate $-\delta V$ where $\delta =1/T$ and *T* is the latency period from the infection to the lysis.

Let *V*(*x*, *t*) denote the density of free lytic viruses in the water column. By Assumption (A_3_), its increase is from the lysis release of infected phytoplankton with the lytic virus replication factor *q*, also known as “burst size" (Beretta and Kuang 1998; Edwards and Steward 2018; Fuhrman et al. 2011). The reduction in *V* is caused by death with a rate $-\mu _v V$ and infectious consumption with a rate $-\beta SV$. The free lytic virus particles move randomly in the water column under the influence of turbulence with a diffusion rate $d_v$. Neumann boundary conditions at $x=0$ and $x=x_l$ mean that no free lytic virus particles enter or leave the water column.

The function *N*(*x*, *t*) describes dissolved nutrient concentration in the water column. The nutrient supply is through the nutrient exchange at the bottom of the water column ($x=x_l$) with a fixed nutrient input concentration $N^0$ and an exchange rate $\alpha$ (Klausmeier and Litchman 2001; Ryabov et al. 2010; Yoshiyama et al. 2009; Zhang et al. 2018). The nutrients are transported by turbulence in the water column with a diffusion rate $d_n$. A no-flux boundary condition is imposed at $x=0$ since there is no nutrient exchange at the water surface. The nutrient consumption in the water column by susceptible and virus-infected phytoplankton is described by a rate $-c_prg(N)f(L(x,S+\theta I))(S+\theta I)$.

The above assumptions and analysis yield the following complete model for phytoplankton-virus interactions with light and nutrients

2.1 $$\begin{aligned} \begin{aligned} S_t&=\underbrace{d_pS_{xx}-\omega S_x}\limits _{\text {diffusion and advection}}+\underbrace{rg(N)f(L(x,S+\theta I))S}\limits _{\text {susceptible phytoplankton growth}}-\underbrace{\mu _p S-\eta (S+\theta I)S}\limits _{\text {susceptible phytoplankton loss}}-\underbrace{b\beta SV}\limits _{\text {infection}},\\ I_t&=\underbrace{d_pI_{xx}-\omega I_x}\limits _{\text {diffusion and advection}}+ \underbrace{b\beta SV}\limits _{\text {infection}}-\underbrace{\delta I}\limits _{\text {lysis death of virus-infected phytoplankton}},\\ V_t&=\underbrace{d_vV_{xx}}\limits _{\text {diffusion}}+\underbrace{q\delta I}\limits _{\text {lysis release of virus-infected phytoplankton}}-\underbrace{\mu _vV}\limits _{\text {free lytic virus death}}-\underbrace{\beta SV}\limits _{\text {infection}},\\ N_t&=\underbrace{d_nN_{xx}}\limits _{\text {diffusion}}- \underbrace{c_prg(N)f(L(x,S+\theta I))(S+\theta I)}\limits _{\text {phytoplankton consumption}}, \end{aligned} \end{aligned}$$

for $x\in (0,x_l)$ and $t>0$ with the boundary conditions

2.2 $$\begin{aligned} \begin{aligned}&d_pS_x(0,t)-\omega S(0,t)=d_pS_x(x_l,t)-\omega S(x_l,t)=0,~t>0,\\&d_pI_x(0,t)-\omega I(0,t)=d_pI_x(x_l,t)-\omega I(x_l,t)=0,~t>0,\\&V_x(0,t)=V_x(x_l,t)=0,~t>0,\\&N_x(0,t)=0,~d_nN_x(x_l,t)=\alpha (N^0-N(x_l,t))~\text {(nutrient exchange)},~t>0 \end{aligned} \end{aligned}$$

and the initial conditions

2.3 $$\begin{aligned} \begin{aligned}&S(x,0)=S_0(x)\ge \not \equiv 0,~I(x,0)=I_0(x)\ge \not \equiv 0, \; x\in (0,x_l),\\&V(x,0)=V_0(x)\ge \not \equiv 0,~N(x,0)=N_0(x)\ge \not \equiv 0, \; x\in (0,x_l). \end{aligned} \end{aligned}$$

Here $\omega \in {\mathbb {R}}$, $\theta \in [0,1]$, and remaining parameters are assumed to be positive constants.

Model (2.1) is a system of four reaction–diffusion–advection equations with a nonlocal structure. To explore the phytoplankton-virus interactions, we rigorously analyze the dynamic properties of model (2.1) including basic properties and behavior of solutions and existence and stability of nonnegative steady states.

## Model analysis

The main purpose of this section is to explore the theoretical results of model (2.1)–(2.3). Some basic properties of solutions of model (2.1)–(2.3) are given in Sect. 3.1. The study of nonnegative steady state solutions is in Sect. 3.2. The dynamic numerical simulations are performed to explain and supplement our theoretical results in Sect. 3.3.

### Basic properties of solutions

Let $X=C([0,x_l],{\mathbb {R}}^4)$ denote the Banach space of all continuous functions defined on $[0,x_l]$ with values in ${\mathbb {R}}^4$ and the norm being the supremum norm. The feasible domain ${\mathcal {W}}$ for (2.1)–(2.3) is the positive cone in *X*:

3.1 $$\begin{aligned} {\mathcal {W}}:=\{(S,I,V,N)\in C([0,x_l],{\mathbb {R}}^4):S(\cdot )\ge 0,I(\cdot )\ge 0,V(\cdot )\ge 0,N(\cdot )\ge 0\}.\nonumber \\ \end{aligned}$$

#### Theorem 3.1

The system (2.1)–(2.3) possesses a unique classical solution in ${\mathcal {W}}$ for all $t>0$ and it is dissipative.

#### Proof

The local existence and uniqueness of nonnegative classical solutions of system (2.1)–(2.3) follow from standard arguments (see Martin and Smith 1990). To obtain the global existence of the solutions, we only need to prove that the solutions of system (2.1)–(2.3) are dissipative.

From the *N*-equation in (2.1), we obtain

$$\begin{aligned}{} & {} N_t\le d_nN_{xx},~x\in (0,x_l),~t>0,~N_x(0,t)=0,\\{} & {} d_nN_x(x_l,t)=\alpha (N^0-N(x_l,t)),~t>0. \end{aligned}$$

This implies that

3.2 $$\begin{aligned} \limsup \limits _{t\rightarrow \infty } N(x,t)\le N^0~\text {on}~[0,x_l]. \end{aligned}$$

Let ${\hat{S}} =Se^{-(\omega /d_p)x}$. From the *S*-equation, we have

$$\begin{aligned}&{\hat{S}}_t\le d_p{\hat{S}}_{xx}+\omega {\hat{S}}_x+rf(L^0) {\hat{S}}-\eta {\hat{S}}^2e^{(\omega /d_p)x},~x\in (0,x_l),~t>0,\\&{\hat{S}}_x(0,t)={\hat{S}}_x(x_l,t)=0,~t>0. \end{aligned}$$

If $$\omega >0$$, then

$$\begin{aligned} \limsup \limits _{t\rightarrow \infty } S(x,t)=\limsup \limits _{t\rightarrow \infty }{\hat{S}}(x,t)e^{(\omega /d_p)x}\le \displaystyle \frac{rf(L^0)e^{(\omega /d_p)x_l}}{\eta } ~\text {on}~[0,x_l]. \end{aligned}$$

If $$\omega <0$$, then

$$\begin{aligned} \limsup \limits _{t\rightarrow \infty } S(x,t)=\limsup \limits _{t\rightarrow \infty }{\hat{S}}(x,t)e^{(\omega /d_p)x}\le \displaystyle \frac{rf(L^0)e^{(-\omega /d_p)x_l}}{\eta } ~\text {on}~[0,x_l]. \end{aligned}$$

Hence

3.3 $$\begin{aligned} \limsup \limits _{t\rightarrow \infty } S(x,t)\le \displaystyle \frac{rf(L^0)e^{(|\omega |/d_p)x_l}}{\eta } ~\text {on}~[0,x_l]. \end{aligned}$$

From (3.3), for any $\epsilon >0$, there exists a $t_1>0$ such that $S(x,t)\le rf(L^0)e^{(|\omega |/d_p)x_l}/\eta +\epsilon$ on $[0,x_l]$ for any $t\ge t_1$. Let ${\hat{I}} =Ie^{-(\omega /d_p)x}$. Adding the *S*-equation and the *I*-equation gives

$$\begin{aligned} \begin{aligned} ({\hat{S}}+{\hat{I}})_t&\le d_p({\hat{S}}+{\hat{I}})_{xx}+\omega ({\hat{S}}+{\hat{I}})_x+rf(L^0)\left( \displaystyle \frac{rf(L^0) e^{(|\omega |/d_p)x_l}}{\eta }+\epsilon \right) e^{-(\omega /d_p)x}\\&\quad -\min \{\mu _p,\delta \}({\hat{S}}+{\hat{I}}) \end{aligned} \end{aligned}$$

for $x\in (0,x_l)$ and $t>t_1$ with the boundary condition

$$\begin{aligned} ({\hat{S}}+{\hat{I}})_x(0,t)=({\hat{S}}+{\hat{I}})_x(x_l,t)=0,~t>t_1. \end{aligned}$$

Applying the parabolic comparison theorem, we get

3.4 $$\begin{aligned} \begin{aligned} \limsup \limits _{t\rightarrow \infty } I(x,t)&\le \limsup \limits _{t\rightarrow \infty } (S+I)(x,t)= \limsup \limits _{t\rightarrow \infty } ({\hat{S}}+{\hat{I}})e^{(\omega /d_p)x}\\&\le \displaystyle \frac{\left( rf(L^0)\right) ^2 e^{2(|\omega |/d_p)x_l}}{\eta \min \{\mu _p,\delta \}} ~\text {on}~[0,x_l]. \end{aligned} \end{aligned}$$

For the $\epsilon >0$ above, there exists a $t_2>0$ such that

$$\begin{aligned} I(x,t)\le \displaystyle \frac{\left( rf(L^0)\right) ^2 e^{2(|\omega |/d_p)x_l}}{\eta \min \{\mu _p,\delta \}}+\epsilon ~\text {on}~[0,x_l] \end{aligned}$$

for any $t\ge t_2$. From the *V*-equation in (2.1), we have

$$\begin{aligned}&V_t\le d_vV_{xx}+q\delta \left( \displaystyle \frac{\left( rf(L^0)\right) ^2 e^{2(|\omega |/d_p)x_l}}{\eta \min \{\mu _p,\delta \}}+\epsilon \right) -\mu _vV,~ x\in (0,x_l),~t>t_2,\\&V_x(0,t)=V_x(x_l,t)=0,~t>t_2. \end{aligned}$$

Hence,

3.5 $$\begin{aligned} \limsup \limits _{t\rightarrow \infty }V(x,t)\le \displaystyle \frac{q\delta \left( rf(L^0)\right) ^2 e^{2(|\omega |/d_p)x_l}}{\eta \mu _v\min \{\mu _p,\delta \}}~\text {on}~[0,x_l]. \end{aligned}$$

Combining (3.2)–(3.5) shows that the solutions of system (2.1)–(2.3) are dissipative. This completes the proof. $\square$

#### Remark 3.2

By Theorem 3.1, there exists a semiflow $\Theta (t):{\mathcal {W}}\rightarrow {\mathcal {W}}$ for (2.1)–(2.3) satisfying

$$\begin{aligned} \Theta (t)(\sigma _0)(x)=(S(x,t,\sigma _0),I(x,t,\sigma _0),V(x,t,\sigma _0),N(x,t,\sigma _0)),~x\in [0,x_l],~t\ge 0 \end{aligned}$$

for every $\sigma _0=(S_0,I_0,V_0,N_0)\in {\mathcal {W}}$. It follows from the dissipativeness of the solutions for (2.1)–(2.3) that $\Theta (t)$ is point dissipative. Furthermore, there exists a global compact attractor for (2.1)–(2.3) in ${\mathcal {W}}$ from Theorem 3.4.8 in Hale (1988) since $\Theta (t)$ is compact.

### Steady states

In order to investigate the spread of lytic viruses among phytoplankton, we explore nonnegative steady states of model (2.1)–(2.3). There are three types of steady state solutions of model (2.1)–(2.3) as follows.

(i): Extinction steady state $E_1=(0,0,0,N^0)$.
(ii): Disease-free steady state $E_2=(S_2(x),0,0,N_2(x))$, where $S_2(x)$ and $N_2(x)$ satisfy
  3.6 $$\begin{aligned} \begin{aligned}&d_pS''-\omega S'+rg(N)f(L(\cdot ,S))S-\mu _p S-\eta S^2=0,~x\in (0,x_l),\\&d_nN''-c_prg(N)f(L(\cdot ,S))S=0,~x\in (0,x_l),\\&d_pS'(0)-\omega S(0)=d_pS'(x_l)-\omega S(x_l)=0,\\&N'(0)=0,~d_nN'(x_l)=\alpha (N^0-N(x_l)). \end{aligned} \end{aligned}$$
(iii): Endemic steady state $E_3=(S_3(x),I_3(x),V_3(x),N_3(x))$, where $S_3(x),I_3(x),V_3(x)$ and $N_3(x)$ satisfy
  3.7 $$\begin{aligned} \begin{aligned}&d_pS''-\omega S'+rg(N)f(L(\cdot ,S+\theta I))S-\mu _p S-\eta (S+\theta I)S-b\beta SV=0,\\&d_pI''-\omega I'+b\beta SV-\delta I=0,\\&d_vV''+q\delta I-\mu _vV-\beta SV=0,\\&d_nN''-c_prg(N)f(L(\cdot ,S+\theta I))(S+\theta I)=0 \end{aligned} \end{aligned}$$
  on $(0,x_l)$ with the boundary conditions
  3.8 $$\begin{aligned} \begin{aligned}&d_pS'(0)-\omega S(0)=d_pS'(x_l)-\omega S(x_l)=0,\\&d_pI'(0)-\omega I(0)=d_pI'(x_l)-\omega I(x_l)=0,\\&V'(0)=V'(x_l)=0,~N'(0)=0,~d_nN'(x_l)=\alpha (N^0-N(x_l)). \end{aligned} \end{aligned}$$

To characterize the dynamic behavior of model (2.1)–(2.3), we define the basic ecological reproductive index for phytoplankton invasion. For $h\in L^\infty ([0,x_l])$, let $\lambda _1(d_p,\omega ,x_l,h(x))$ denote the principal eigenvalue of

3.9 $$\begin{aligned} \begin{aligned}&d_p\phi ''(x)-\omega \phi '(x)+h(x)\phi (x)=\lambda \phi (x),~x\in (0,x_l),\\&d_p\phi '(0)-\omega \phi (0)=d_p\phi '(x_l)-\omega \phi (x_l)=0. \end{aligned} \end{aligned}$$

By Proposition 3.1 in Wang et al. (2019), $\lambda _1$ exists and it is unique. Moreover, $\lambda _1(d_p,\omega ,x_l,h_1(x))\ge \lambda _1(d_p,\omega ,x_l,h_2(x))$ if $h_1(x)\ge h_2(x)$ on $[0,x_l]$. The basic ecological reproductive index for phytoplankton invasion is defined as

3.10 $$\begin{aligned} \begin{aligned} R_p=\displaystyle \frac{\mu _p^*}{\mu _p},~~\text { where } \;\; \mu _p^*=\lambda _1(d_p,\omega ,x_l,rg(N^0)f(L(x,0))). \end{aligned} \end{aligned}$$

Note that $\mu _p^*$ is related to the stability of the unique extinction steady state $E_1$ (see (3.11)). The index $R_p$ measures the reproductive capacity of phytoplankton, and it describes the average number of new phytoplankton cells produced by per cubic meter of phytoplankton in a life cycle.

#### Theorem 3.3

$E_1\equiv (0,0,0,N^0)$ is the unique extinction steady state of (2.1)–(2.3). If $R_p<1$, then $E_1$ is globally asymptotically stable, while $E_1$ is unstable if $R_p>1$.

#### Proof

It is clear that $E_1\equiv (0,0,0,N^0)$ exists uniquely. The local stability of $E_1$ is determined by the eigenvalue problem

3.11 $$\begin{aligned} \begin{aligned}&\lambda \xi =d_p\xi ''(x)-\omega \xi '(x)+ \left( rg(N^0)f(L(x,0))-\mu _p\right) \xi ,~x\in (0,x_l),\\&\lambda \varphi =d_p\varphi ''(x)-\omega \varphi '(x)-\delta \varphi ,~x\in (0,x_l),\\&\lambda \psi =d_v\psi ''(x)+q\delta \varphi -\mu _v\psi ,~x\in (0,x_l),\\&\lambda \zeta =d_n\zeta ''(x)-c_prg(N^0)f(L(x,0))(\xi +\theta \varphi ),~x\in (0,x_l) \end{aligned} \end{aligned}$$

with the boundary conditions

3.12a $$\begin{aligned}&d_p\xi '(0)-\omega \xi (0)=d_p\xi '(x_l)-\omega \xi (x_l)=0,\end{aligned}$$

3.12b $$\begin{aligned}&d_p\varphi '(0)-\omega \varphi (0)=d_p\varphi '(x_l)-\omega \varphi (x_l)=0,\end{aligned}$$

3.12c $$\begin{aligned}&{\psi '(0)=\psi '(x_l)=0},\end{aligned}$$

3.12d $$\begin{aligned}&\zeta '(0)=0,~d_n\zeta '(x_l)=-\alpha \zeta (x_l). \end{aligned}$$

One can observe that $\lambda$ is an eigenvalue of (3.11) if and only if $\lambda$ is an eigenvalue of one of the following four operators

$$\begin{aligned}&d_p\displaystyle \frac{d^2}{dx^2}-\omega \displaystyle \frac{d}{dx}+\left( rg(N^0)f(L(\cdot ,0))-\mu _p\right) ,\\&d_p\displaystyle \frac{d^2}{dx^2}-\omega \displaystyle \frac{d}{dx}-\delta ,~d_v\displaystyle \frac{d^2}{dx^2}-\mu _v,~d_n\displaystyle \frac{d^2}{dx^2} \end{aligned}$$

with the boundary conditions (3.12a)–(3.12d) (see Theorem 4.1 in Nie et al. 2017). Note that all eigenvalues of the operators $d_v\displaystyle \frac{d^2}{dx^2}-\mu _v$ with the Neumann boundary condition (3.12c) and $d_n\displaystyle \frac{d^2}{dx^2}$ with the Robin boundary condition (3.12d) are less than 0. By (3.9), all eigenvalues of the operator $d_p\displaystyle \frac{d^2}{dx^2}-\omega \displaystyle \frac{d}{dx}-\delta$ with (3.12b) are less than 0. Applying (3.9) again, all eigenvalues of the operator $d_p\displaystyle \frac{d^2}{dx^2}-\omega \displaystyle \frac{d}{dx}+\left( rg(N^0)f(L(\cdot ,0))-\mu _p\right)$ with (3.12a) are less than 0 if $R_p<1$, and it has at least one eigenvalue greater than 0 if $R_p>1$. The above analysis shows that $E_1$ is locally asymptotically stable when $R_p<1$, and $E_1$ is unstable when $R_p>1$.

To obtain the global stability of $E_1$ when $R_p<1$, we only need to prove that it is globally attractive. For any $\epsilon >0$, from (3.2) and (3.5), there exists a $t_1^*>0$ such that $N(\cdot ,t)\le N^0+\epsilon$ and $V(\cdot ,t)\le q\delta \left( rf(L^0)\right) ^2 e^{2(|\omega |/d_p)x_l}/(\eta \mu _v\min \{\mu _p,\delta \})+\epsilon$ for $t\ge t_1^*$. Let $\xi$ be the first component of the positive eigenfunction of (3.11) corresponding to $\mu =\mu _p^*$ satisfying $S(x,t_1^*)\le c\xi (x)$ for some $c>0$. Let ${\hat{S}} =Se^{-(\omega /d_p)x}$ and ${\hat{\xi }}=\xi e^{-(\omega /d_p)x}$. It follows from the *S*-equation in model (2.1) that

$$\begin{aligned}&{\hat{S}}_t\le d_p{\hat{S}}_{xx}+\omega {\hat{S}}_x +rg(N^0+\epsilon )f(L(x,0)){\hat{S}}-\mu _p{\hat{S}},~x\in (0,x_l),~t> t_1^*,\\&{\hat{S}}_x(0,t)={\hat{S}}_x(x_l,t)=0,~t>t_1^*. \end{aligned}$$

By the comparison theorem of parabolic systems, we have

$$\begin{aligned} {\hat{S}}(x,t)\le ce^{-(\mu _p-\lambda _1(d_p,\omega ,x_l,rg(N^0+\epsilon )f(L(x,0))))(t-t_1^*)}\hat{\xi }(x),~x\in [0,x_l],~\text {for any}~t\ge t_1^*. \end{aligned}$$

This shows that $\limsup \limits _{t\rightarrow \infty }S(x,t)=\limsup \limits _{t\rightarrow \infty }{\hat{S}}(x,t)e^{(\omega /d_p)x}=0$ on $[0,x_l]$ since $R_p<1$ and $\epsilon$ is sufficiently small. Hence we can find a $t_2^*>t_1^*$ satisfying $S(x,t)\le \epsilon$ on $[0,x_l]$ for any $t\ge t_2^*$. Let ${\hat{I}} =Ie^{-(\omega /d_p)x}$. It follows that

$$\begin{aligned}&{\hat{I}}_t\le d_p{\hat{I}}_{xx} +\omega {\hat{I}}_x +b\beta \epsilon \left( \displaystyle \frac{q\delta \left( rf(L^0)\right) ^2 e^{2(|\omega |/d_p)x_l}}{\eta \mu _v\min \{\mu _p,\delta \}}+\epsilon \right) \\&e^{-(\omega /d_p)x}-\delta {\hat{I}},~x\in (0,x_l),~t> t_2^*,\\&{\hat{I}}_x(0,t)={\hat{I}}_x(x_l,t)=0,~t>t_2^*. \end{aligned}$$

Then

3.13 $$\begin{aligned} \limsup \limits _{t\rightarrow \infty }I(x,t)=\limsup \limits _{t\rightarrow \infty }{\hat{I}}(x,t)e^{(\omega /d_p)x}=0~\text {on}~[0,x_l] \end{aligned}$$

since $\epsilon$ is sufficiently small. Similarly, we can also obtain

3.14 $$\begin{aligned} \limsup \limits _{t\rightarrow \infty }V(x,t)=0~\text {on}~[0,x_l]. \end{aligned}$$

Following Theorem 1.8 in Mischaikow et al. (1995) or Theorem 4.1 in Thieme (1992), the *N*-equation in (2.1) becomes

$$\begin{aligned} N_t=d_nN_{xx},~x\in (0,x_l), ~N_x(0,t)=0,~d_nN_x(x_l,t)=\alpha (N^0-N(x_l,t)) \end{aligned}$$

for sufficiently large *t*. Thus

3.15 $$\begin{aligned} \lim \limits _{t\rightarrow \infty }N(x,t)=N^0~\text {on}~[0,x_l], \end{aligned}$$

and then $E_1$ is globally attractive. $\square$

#### Remark 3.4

1. The basic ecological reproductive index for phytoplankton invasion $R_p$ measures the viability of phytoplankton. $R_p<1$ means that phytoplankton go extinct and nutrients are evenly distributed in the water column. $\mu _p=\mu _p^*$ is a critical loss rate that determines whether phytoplankton can invade an aquatic ecosystem.
2. From the structure of (3.10), the basic ecological reproductive index $R_p$ depends on important ecological parameters such as spatial factors $d_p,\omega$, the water column depth $x_l$, nutrient concentration at the bottom of the water column $N^0$ and light intensity at the water surface $L^0$. According to Theorems 3.2 and 3.4 in Hsu and Lou (2010), we conclude that $dR_p/d\omega <0$ and $dR_p/dx_l<0$. By the monotonicity of $\lambda _1$ with respect to $rg(N^0)f(L(x,0))$, $dR_p/dN^0>0$ and $dR_p/dL^0>0$. Thus, high $N^0$ and $L^0$ are conducive to phytoplankton invasion, while high $\omega$ and $x_l$ prevent phytoplankton invasion. The dependence of $R_p$ on $d_p$ is complicated. Both high and low $d_p$ may be detrimental to the survival of phytoplankton (see Huisman et al. 2002).

When $R_p>1$, the extinction state $E_1$ is unstable and phytoplankton can persist. To establish the existence of $E_2$ which has a positive phytoplankton mass, we first derive *a priori* estimate for nonnegative solutions of (3.6).

#### Lemma 3.5

Suppose $(S_2(x), N_2(x))$ is a nonnegative solution of (3.6) with $S_2, N_2\not \equiv 0$. Then $0<N_2(x)<N^0$, $0<S_2(x)\le ((rg(N^0)f(L^0)+\mu _p)e^{(|\omega |/d_p)x_l})/\eta$ on $[0,x_l]$ and $0<\mu _p<\mu _p^*$.

#### Proof

Let $\bar{U}(x)=e^{-(\omega /d_p)x}S_2(x)$. Then $\bar{U}$ satisfies

$$\begin{aligned}&-d_p\bar{U}''-\omega \bar{U}'+\left( \mu _p+\eta \bar{U}e^{(\omega /d_p)x}\right) \bar{U}=rg(N_2)f(L(x,S_2))\bar{U}\ge 0,~x\in (0,x_l),\\&\bar{U}'(0)=\bar{U}'(x_l)=0. \end{aligned}$$

From the strong maximum principle and Hopf boundary lemma, we have $S_2(x)=\bar{U}(x)e^{(\omega /d_p)x}>0$ on $[0,x_l]$. It follows from (3.6) that

$$\begin{aligned} \alpha (N^0-N_2(x_l))&=\int _0^{x_l} c_prg(N_2(x))f(L(x,S_2(x)))S_2(x)dx\\&=c_p\displaystyle \int _0^{x_l}\left( \mu _p S_2(x)+\eta S_2^2(x)\right) dx>0, \end{aligned}$$

and then $N_2(x_l)<N^0$. By the *N*-equation in (3.6), we obtain

$$\begin{aligned}&-d_nN_2''+\left( c_prf(L(x,S_2))S_2\displaystyle \int _0^1g'(sN_2)ds\right) N_2=0,~x\in (0,x_l),\\&N_2'(0)=0,~d_nN_2'(x_l)=\alpha (N^0-N_2(x_l))>0. \end{aligned}$$

This implies that $N_2(x)>0$ on $[0,x_l]$ from the maximum principle. Note that

$$\begin{aligned} d_nN_2''=c_prg(N_2)f(L(x,S_2))S_2>0,~x\in (0,x_l). \end{aligned}$$

Combining its boundary conditions give $N'_2(x)>0$ on $(0,x_l)$. Thus, $0<N_2<N^0$ on $[0,x_l]$.

From the *S*-equation in (3.6) and $S_2>0$, we get

$$\begin{aligned} \mu _p= & {} \lambda _1(d_p,\omega ,x_l,rg(N_2)f(L(x,S_2))-\eta S_2)\\< & {} \lambda _1(d_p,\omega ,x_l,rg(N^0)f(L(x,0)))=\mu _p^*. \end{aligned}$$

By applying the similar arguments of Lemma 2.2 in Pang et al. (2019), we can conclude that $S_2(x)\le ((rg(N^0)f(L^0)+\mu _p)e^{(|\omega |/d_p)x_l})/\eta$ on $[0,x_l]$. $\square$

We now prove the existence of disease-free steady state $E_2$ by showing $E_2$ bifurcating from the line of extinction state $\{(\mu _p,E_1):\mu _p>0\}$ at $\mu _p=\mu _p^*$.

#### Theorem 3.6

(i): If $R_p>1$ (equivalently, $\mu _p\in (0,\mu _p^*)$), then (2.1)–(2.3) has at least one disease-free steady state $E_2$.
(ii): There exists an $\varepsilon >0$ such that for each given $s\in (0,\varepsilon )$ the bifurcating solution $(\mu _p(s),S_2(s,\cdot ),N_2(s,\cdot ))$ is locally asymptotically stable with respect to the susceptible phytoplankton-nutrient model
  3.16 $$\begin{aligned} \begin{aligned}&S_t=d_pS_{xx}-\omega S_x+rg(N)f(L(x,S))S -\mu _pS-\eta S^2, ~x\in (0,x_l),~t>0,\\&N_t=d_nN_{xx}-c_prg(N)f(L(x,S))S,~x\in (0,x_l), ~t>0,\\&d_pS_x(0,t)-\omega S(0,t)=d_pS_x(x_l,t)-\omega S(x_l,t)=0,~t>0,\\&N_x(0,t)=0,~d_nN_x(x_l,t)=\alpha (N^0-N(x_l,t)),~t>0. \end{aligned} \end{aligned}$$

#### Proof

(i) The first part of the proof is divided into two steps. The first one is to obtain the existence of local bifurcation of $E_2$. Applying the Crandall–Rabinowitz bifurcation theorem (see Theorem 1.7 in Crandall and Rabinowitz 1971), we show that there is a positive solution branch $\Pi _2^+=\{(\mu _p(s),S_2(s,\cdot ),N_2(s,\cdot )):~0<s<\varepsilon \}$ for some $\varepsilon >0$ from $\Pi _1=\{(\mu _p,0,N^0): \mu _p>0\}$ at $\mu _p=\mu _p^*$. The second is to explore the global bifurcation structure of $E_2$. That is to show that the disease-free steady state $E_2$ exists for all $\mu _p\in (0,\mu _p^*)$ by using Theorem 3.3 and Remark 3.4 in Shi and Wang (2009).

Let $\tilde{S}=Se^{-(\omega /d_p)x}$ and $\tilde{N}=N^0-N$. Then (3.6) is transformed into

3.17 $$\begin{aligned} \begin{aligned}&d_p\tilde{S}''+\omega \tilde{S}'+rg(N^0-\tilde{N})f(L(x,\tilde{S}e^{(\omega /d_p)x}))\tilde{S}\\&-\mu _p \tilde{S}-\eta \tilde{S}^2e^{(\omega /d_p)x}=0,~x\in (0,x_l),\\&-d_n\tilde{N}''-c_prg(N^0-\tilde{N})f(L(x,\tilde{S}e^{(\omega /d_p)x}))\tilde{S}e^{(\omega /d_p)x}=0,~x\in (0,x_l),\\&\tilde{S}'(0)=\tilde{S}'(x_l)=0,~\tilde{N}'(0)=d_n\tilde{N}'(x_l)+\alpha \tilde{N}(x_l)=0 \end{aligned} \end{aligned}$$

and the extinction state $(S,N)=(0,N^0)$ is transformed to $(\tilde{S},\tilde{N})=(0,0)$. Set ${\mathbb {W}}:={\mathbb {W}}_1\times {\mathbb {W}}_2$, where

$$\begin{aligned} \begin{aligned} {\mathbb {W}}_1&:=\{h\in W^{2,p}(0,x_l):h'(0)=h'(x_l)=0\},\\ {\mathbb {W}}_2&:=\{h\in W^{2,p}(0,x_l):h'(0)=d_nh'(x_l)+\alpha h(x_l)=0\}. \end{aligned} \end{aligned}$$

**Step 1** (Local bifurcation). Define $T:{\mathbb {R}}^+\times {\mathbb {W}}\rightarrow L^p(0,x_l)\times L^p(0,x_l), p>1$ as follows

$$\begin{aligned} \begin{aligned} T(\mu _p,\tilde{S},\tilde{N}) =\begin{pmatrix} d_p\tilde{S}''+\omega \tilde{S}'+rg(N^0-\tilde{N})f(L(x,\tilde{S}e^{(\omega /d_p)x}))\tilde{S}-\mu _p \tilde{S}-\eta \tilde{S}^2e^{(\omega /d_p)x}\\ -d_n\tilde{N}''-c_prg(N^0-\tilde{N})f(L(x,\tilde{S}e^{(\omega /d_p)x}))\tilde{S}e^{(\omega /d_p)x} \end{pmatrix}. \end{aligned} \end{aligned}$$

It can be directly observed that $T(\mu _p,0,0)=0$ for $\mu _p>0$. Let $Q:=T_{(\tilde{S},\tilde{N})}(\mu _p^*,0,0)$. A direct calculation shows that

$$\begin{aligned} \begin{aligned} Q[\xi ,\zeta ] =\begin{pmatrix} d_p\xi ''+\omega \xi '+\left( rg(N^0)f(L(x,0))-\mu _p^*\right) \xi \\ -d_{n}\zeta ''-c_prg(N^0)f(L(x,0))\xi e^{(\omega /d_p)x} \end{pmatrix} \end{aligned} \end{aligned}$$

for any $$(\xi ,\zeta )\in {\mathbb {W}}$$.

We claim that *Q* is a Fredholm operator with index zero. Following the proof of Theorem 3.5 in Yan et al. (2022), we have $\dim \ker Q=1$ and $\ker Q={{\,\textrm{span}\,}}\{(\bar{\xi },\bar{\zeta })\}$ with $\bar{\xi }>0, \bar{\zeta }>0$ on $[0,x_l]$. Here $\bar{\xi }\in {\mathbb {W}}_1$ satisfies

3.18 $$\begin{aligned} d_p\bar{\xi }''+\omega \bar{\xi }'+\left( rg(N^0)f(L(x,0))-\mu _p^*\right) \bar{\xi }=0,~x\in (0,x_l), \end{aligned}$$

and $\bar{\zeta }\in {\mathbb {W}}_2$ can be uniquely solved by

$$\begin{aligned} -d_{n}\zeta ''-c_prg(N^0)f(L(x,0)){\bar{\xi }} e^{(\omega /d_p)x} =0,~x\in (0,x_l). \end{aligned}$$

If $(\kappa _1,\kappa _2)\in {{\,\textrm{range}\,}}Q$, then we can find $({\hat{\xi }},{\hat{\zeta }})\in {\mathbb {W}}$ satisfying

3.19 $$\begin{aligned} \begin{aligned}&d_p{\hat{\xi }}''+\omega {\hat{\xi }}'+\left( rg(N^0)f(L(x,0))-\mu _p^*\right) {\hat{\xi }}=\kappa _1,~x\in (0,x_l),\\&-d_{n}{\hat{\zeta }}''-c_prg(N^0)f(L(x,0)){\hat{\xi }} e^{(\omega /d_p)x}=\kappa _2,~x\in (0,x_l). \end{aligned} \end{aligned}$$

By (3.18) and the first equation in (3.19), we have $\displaystyle \int _{0}^{x_l}\kappa _1(x)e^{(\omega /d_p)x}{\bar{\xi }}(x)dx=0$, and $\hat{\xi }\in {\mathbb {W}}_1$ can be uniquely solved under this condition. It follows from the Fredholm alternative theorem that ${\hat{\zeta }\in } {\mathbb {W}}_2$ can then be uniquely solved by the second equation in (3.19). Hence,

$$\begin{aligned} {{\,\textrm{range}\,}}Q=\left\{ (\kappa _1,\kappa _2)\in L^p(0,x_l)\times L^p(0,x_l):\displaystyle \int _{0}^{x_l}\kappa _1(x)e^{(\omega /d_p)x}{\bar{\xi }}(x)dx=0\right\} \end{aligned}$$

and ${{\,\textrm{codim}\,}}{{\,\textrm{range}\,}}Q=1$. This implies that *Q* is a Fredholm operator with index zero.

One can observe that $T_{(\mu _p,(\tilde{S},\tilde{N}))}(\mu _p^*,0,0) (\bar{\xi }, \bar{\zeta })=(-\bar{\xi },0,0)$ and $\displaystyle \int _0^{x_l}e^{(\omega /d_p)x}\bar{\xi }^2(x)dx\not =0$. Then $T_{(\mu _p,(\tilde{S},\tilde{N}))}(\mu _p^*,0,0)\not \in {{\,\textrm{range}\,}}Q$. According to the Crandall–Rabinowitz bifurcation theorem (Theorem 1.7 in Crandall and Rabinowitz (1971)), there is a smooth curve ${\tilde{\Pi }}_2=\{(\mu _p(s),\tilde{S}(s,\cdot ), \tilde{N}(s,\cdot )):~-\varepsilon<s<\varepsilon \}$ for some $\varepsilon >0$ satisfying (3.17) near $(\mu _p^*,0,0)$ with the form

$$\begin{aligned} \tilde{S}(s,\cdot )=s{\bar{\xi }}(\cdot )+o(s),~\tilde{N}(s,\cdot )=s{\bar{\zeta }}(\cdot )+o(s). \end{aligned}$$

Here we also define

$$\begin{aligned}{} & {} \tilde{\Pi }_2^+=\{(\mu _p(s),\tilde{S}(s,\cdot ),\tilde{N}(s,\cdot )):~0<s<\varepsilon \},\\{} & {} \quad \tilde{\Pi }_2^-=\{(\mu _p(s),\tilde{S}(s,\cdot ),\tilde{N}(s,\cdot )):~-\varepsilon<s<0\}. \end{aligned}$$

Let $S_2(s,x)=\tilde{S}(s,x) e^{(\omega /d_p)x}$, $N_2(s,x)=N^0-\tilde{N}(s,x)$ on $[0,x_l]$, and define

$$\begin{aligned}{} & {} \Pi _2^+=\{(\mu _p(s),S_2(s,\cdot ),N_2(s,\cdot )):~0<s<\varepsilon \},\\{} & {} \quad \Pi _2^-=\{(\mu _p(s),S_2(s,\cdot ),N_2(s,\cdot )):~-\varepsilon<s<0\}. \end{aligned}$$

Then the bifurcating branch $\Pi _2^+$ consists of positive solutions of (3.6) as ${\bar{\xi }}>0$ and $N^0>0$.

Let

$$\begin{aligned} \pi (x,{\bar{\xi }},{\bar{\zeta }})=-\displaystyle \frac{hlL(x,0)g(N^0)}{(h+L(x,0))^2}\displaystyle \int _{0}^x{\bar{\xi }}(z)e^{(\omega /d_p)z}dz-\eta {\bar{\xi }} e^{(\omega /d_p)x}-\displaystyle \frac{\gamma f(L(x,0))}{(\gamma +N^0)^2}{\bar{\zeta }}. \end{aligned}$$

Then

$$\begin{aligned} \begin{aligned} \mu _p'(0)&=-\displaystyle \frac{\left\langle \mathcal {L},T_{((\tilde{S},\tilde{N})(\tilde{S},\tilde{N}))}\left( \mu _p^*, 0,0\right) [\bar{\xi },\bar{\zeta }]^2\right\rangle }{2\left\langle \mathcal {L}, T_{(\mu _p,(\tilde{S},\tilde{N}))}\left( \mu _p^*, 0,0\right) [\bar{\xi },\bar{\zeta }]\right\rangle }=\displaystyle \frac{\displaystyle \int _0^{x_l}re^{(\omega /d_p)x} \pi (x,{\bar{\xi }},{\bar{\zeta }})\bar{\xi }^{2}dx}{\displaystyle \int _0^{x_l}e^{(\omega /d_p)x}\bar{\xi }^2dx}<0, \end{aligned} \end{aligned}$$

where $\mathcal {L}$ is a linear functional on $L^p(0,x_l)\times L^p(0,x_l)$ defined by

$$\begin{aligned} \langle \mathcal {L},(\kappa _1, \kappa _2)\rangle = \displaystyle \int _0^{x_l}\kappa _1(x)e^{(\omega /d_p)x}\bar{\xi }dx. \end{aligned}$$

This shows that the bifurcation of $\Pi _2^+$ or $\tilde{\Pi }_2^+$ at $(0,N^0)$ is backward as $\pi <0$.

**Step 2** (Global bifurcation). In view of Theorem 4.3 in Shi and Wang (2009), there exists a connected component $\tilde{\Gamma }$ of $\overline{\Sigma }$ containing ${{\tilde{\Pi }}}_2$ where $\Sigma =\{(\mu _p,\tilde{S},\tilde{N})\in {\mathbb {R}}^+\times {\mathbb {W}}:T(\mu _p,\tilde{S},\tilde{N})=0,(\tilde{S},\tilde{N})\ne (0,0)\}$. Let $\tilde{\Gamma }^+$ be the connected component of $\tilde{\Gamma }{\setminus }\tilde{\Pi }_2^-$ which contains $\tilde{\Pi }_2^+$. It follows from Theorem 4.4 in Shi and Wang (2009) that $\tilde{\Gamma }^+$ satisfies one of the following three alternatives: (1) it is not compact in ${\mathbb {R}}^+ \times {\mathbb {W}}$, (2) it contains another point $({\bar{\mu }}_p,0,0)$ with ${\bar{\mu }}_p\ne \mu _p^*$, (3) it contains a point $(\mu _p,\bar{S}, \bar{N})$ with $0\ne (\bar{S},\bar{N})\in Z$, where *Z* is a closed complement of $\ker Q={{\,\textrm{span}\,}}\{(\bar{\xi },\bar{\zeta })\}$.

Assume that (2) occurs. Then $({\bar{\mu }}_p,0,0)$ is a bifurcation point for $T=0$ with ${\bar{\mu }}_p\ne \mu _p^*$ and 0 is an eigenvalue of $T_{(\tilde{S},\tilde{N})}(\mu _p,0,0)$ and ${\bar{\mu }}_p<\mu _p^*$. There are only finitely many such ${\bar{\mu }}_p$, so without loss of generality we may assume that $\Gamma ^+$ does not contain any other $(\mu _p,0,0)$ with ${\bar{\mu }}_p<\mu _p<\mu _p^*$. Since $\mu _p=\mu _p^*$ is the only value such that $T_{(\tilde{S},\tilde{N})}(\mu _p,0,0)$ has a positive eigenfunction corresponding to a zero eigenvalue, then all solutions of $T(\mu _p, \tilde{S},\tilde{N})=0$ on $\tilde{\Gamma }^+$ near $({\bar{\mu }}_p,0,0)$ must be sign-changing. Since $\tilde{\Gamma }^+ \supseteq \tilde{\Pi }_2^+$, then there exists $(\mu _p, \tilde{S}, \tilde{N})\in \tilde{\Gamma }^+$ which satisfies $\tilde{S}>0$ and $\tilde{N}>0$. From the connectedness of $\Gamma ^+$, there exists $({\hat{\mu _p}}, \tilde{S}, \tilde{N})\in \tilde{\Gamma }^+$ with ${\hat{\mu _p\in }} [{\bar{\mu }}_p,\mu _p^*)$ and $x_0\in [0,x_l]$ such that either $\tilde{S}(x_0)=0$ or $\tilde{N}(x_0)=0$, but $\tilde{S} (x)\ge 0$ and $\tilde{N} (x)\ge 0$ for all $x\in [0,x_l]$. If $\tilde{S}(x_0)=0$, then we also have $\tilde{S}'(x_0)=0$, then we obtain $\tilde{S}\equiv \tilde{N}=0$ on $[0,x_l]$ from the uniqueness of solution to ODE so (2) occurs at ${\hat{\mu _p}}$ and ${\hat{\mu _p}}={\bar{\mu }}_p$. But the solution $({\hat{\mu _p}}, \tilde{S}, \tilde{N})$ is not sign-changing, which is a contradiction. Hence we must have $\tilde{S}(x)>0$ on $[0,x_l]$ and $\tilde{N}(x_0)=0$. But this leads to another contradiction using $\tilde{S} \ge 0$ and maximum principle. Hence (2) cannot occur. Assume that (3) happens. Since $0\ne (\bar{S},\bar{N})\in Z$, then either $\bar{S}$ or $\bar{N}$ is sign-changing as $\bar{\xi }>$ and $\bar{\zeta }>0$. We can follow the similar proof as in the one for alternative (2) to show (3) cannot occurs.

Hence the alternative (1) must occur and the connected component $\tilde{\Gamma }^+$ is not compact in ${\mathbb {R}}^+ \times {\mathbb {W}}$. Moreover the proof in the last paragraph implies that if $(\mu _p, \tilde{S}, \tilde{N})\in \tilde{\Gamma }^+$ then $\tilde{S}>0$ and $\tilde{N}>0$. Let

$$\begin{aligned} \Gamma ^+=\{(\mu _p,S,N):S=\tilde{S} e^{(\omega /d_p)x},N=N^0-\tilde{N},\text{ and }~(\mu _p,\tilde{S},\tilde{N})\in \tilde{\Gamma }^+\}. \end{aligned}$$

Then for any $(\mu _p,S,N)\in \Gamma ^+$, we know that $S=\tilde{S} e^{(\omega /d_p)x}>0$ on $[0,x_l]$, then $N>0$ on $[0,x_l]$ since $N'(0)=0$ and $N''>0$ in $(0,x_l)$. From Lemma 3.5, every positive solution of (3.6) is bounded for $0<\mu _p<\mu _p^*$ and there is no positive solution of (3.6) for $\mu _p\ge \mu _p^*$. Therefore $\Gamma ^+$ can be extended to $\mu _p=0$ and the projection of $\Gamma ^+$ onto $\mu _p$-axis contains $(0,\mu _p^*)$.

(ii) Let ${\mathcal {L}}(\mu _p(s),S(s),N(s))$ be the linearized operator of (3.16) at $(\mu _p(s),S(s),N(s))$. From Corollary 1.13 and Theorem 1.16 in Crandall and Rabinowitz (1973), there exist continuously differentiable functions

$$\begin{aligned}&\gamma _1:[\mu _p^*,\mu _p^*+\varepsilon )\rightarrow {\mathbb {R}},~(\xi _1,\zeta _1):[\mu _p^*,\mu _p^*+\varepsilon )\rightarrow W^{2,p}(0,x_l)\times W^{2,p}(0,x_l),\\&\gamma _2:[0,\varepsilon )\rightarrow {\mathbb {R}},~(\xi _2,\zeta _2):[0,\varepsilon )\rightarrow W^{2,p}(0,x_l)\times W^{2,p}(0,x_l) \end{aligned}$$

such that

$$\begin{aligned}&{\mathcal {L}}(\mu _p,0,N^0)[\xi _1(\mu _p),\zeta _1(\mu _p)]=\gamma _1(\mu _p)[\xi _1(\mu _p),\zeta _1(\mu _p)],\\&{\mathcal {L}}(\mu _p(s),S_2(s,\cdot ),N_2(s,\cdot ))[\xi _2(s),\zeta _2(s)]=\gamma _2(s)[\xi _2(s),\zeta _2(s)] \end{aligned}$$

and

$$\begin{aligned} \lim \limits _{s\rightarrow 0^+}\displaystyle \frac{-s\mu _p'(s)\gamma _1'(\mu _p^*)}{\gamma _2(s)}=1. \end{aligned}$$

Here $\gamma _1(\mu _p^*)=\gamma _2(0)=0$ and $(\xi _1(\mu _p^*),\zeta _1(\mu _p^*))=(\xi _2(0),\zeta _2(0))=({\bar{\xi }},{\bar{\zeta }})$. Note that $\gamma _1(\mu _p)$ is a simple eigenvalue of

$$\begin{aligned}&d_p\xi ''-\omega \xi '+\left( rg(N^0)f(L(x,0))-\mu _p\right) \xi =\gamma _1(\mu _p)\xi ,~x\in (0,x_l),\\&d_p\xi '(0)-\omega \xi (0)=d_p\xi '(x_l)-\omega \xi (x_l)=0. \end{aligned}$$

It follows that $\gamma _1(\mu _p)=\mu _p^*-\mu _p$ and then $\gamma _1'(\mu _p^*)=-1$. Recalling $\mu _p'(0)<0$, we have $\gamma _2(s)<0$ for $s\in (0,\varepsilon )$. By the perturbation theory of linear operators (see [22]), $\gamma _2(s)$ is also the principal eigenvalue of ${\mathcal {L}}(\mu _p(s),S_2(s,\cdot ),N_2(s,\cdot ))$ when *s* is sufficiently small. This means that $(S_2(s,\cdot ),N_2(s,\cdot ))$ is locally asymptotically stable for (3.16). $\square$

In Theorem 3.6, we obtain the existence of $E_2$ for all $0<\mu _p<\mu _p^*$, and the uniqueness and stability of $E_2$ is unknown. Numerical simulations suggest that the disease-free steady state $E_2$ is unique if $R_p>1$.

Next we show that when $R_p>1$ and some additional conditions are satisfied, solutions of model (2.1)–(2.3) are uniformly persistent and there exists at least one endemic steady state $E_3$. To obtain the conclusions, we define the basic reproduction number for lytic virus transmission. Letting $\bar{I}=Ie^{-(\omega /d_p)x}$ and linearizing (2.1) at a disease-free steady state $E_2=(S_2,0,0,N_2)$, we obtain

$$\begin{aligned} \begin{aligned}&{\bar{I}}_t=d_p{\bar{I}}_{xx}+\omega {\bar{I}}_x+b\beta e^{-(\omega /d_p)x}S_2V-\delta {\bar{I}},~x\in (0,x_l),~t>0,\\&V_t=d_vV_{xx}+q\delta e^{(\omega /d_p)x}{\bar{I}}-\mu _vV-\beta S_2V,~x\in (0,x_l),~t>0,\\&{\bar{I}}_x(0,t)={\bar{I}}_x(x_l,t)=V_x(0,t)=V_x(x_l,t)=0,~t>0. \end{aligned} \end{aligned}$$

For $h_1,h_2\in C([0,x_l],{\mathbb {R}}_+)$, we consider the following linear parabolic system

3.20 $$\begin{aligned} \begin{aligned}&{\bar{I}}_t=d_p{\bar{I}}_{xx}+\omega {\bar{I}}_x+b\beta e^{-(\omega /d_p)x}h_1V-\delta {\bar{I}},~x\in (0,x_l),~t>0,\\&V_t=d_vV_{xx}+q\delta e^{(\omega /d_p)x}{\bar{I}}-\mu _vV-\beta h_2V,~x\in (0,x_l),~t>0,\\&{\bar{I}}_x(0,t)={\bar{I}}_x(x_l,t)=V_x(0,t)=V_x(x_l,t)=0,~t>0. \end{aligned} \end{aligned}$$

Let $\Theta ^{h_2}(t):C([0,x_l],{\mathbb {R}}^2)\rightarrow C([0,x_l],{\mathbb {R}}^2)$ denote the solution semigroup generated by the following system

$$\begin{aligned}&{\bar{I}}_t=d_p{\bar{I}}_{xx}+\omega {\bar{I}}_x-\delta {\bar{I}},~x\in (0,x_l),~t>0,\\&V_t=d_vV_{xx}+q\delta e^{(\omega /d_p)x}{\bar{I}}-(\mu _v+\beta h_2)V,~x\in (0,x_l),~t>0,\\&{\bar{I}}_x(0,t)={\bar{I}}_x(x_l,t)=V_x(0,t)=V_x(x_l,t)=0,~t>0. \end{aligned}$$

Define

$$\begin{aligned} F^{h_1}(x)=\begin{pmatrix}0&{}b\beta e^{-(\omega /d_p)x}h_1\\ 0&{}0\end{pmatrix}. \end{aligned}$$

We assume that the distribution of initial infected phytoplankton and free lytic viruses is $\rho (x)=(\rho _1(x),\rho _2(x))$. As time evolves, the distribution at time *t* is $\Theta ^{h_2}(t)\rho (x)$. Therefore, it can be deduced that the distribution of total new infected phytoplankton is

$$\begin{aligned} K^{h_1,h_2}(\rho )(x)=\displaystyle \int _0^\infty F^{h_1}(x)\Theta ^{h_2}(t)\rho (x)dt. \end{aligned}$$

Here $K^{h_1,h_2}$ is called the next generation operator, and its spectral radius is $r(K^{h_1,h_2})$. It follows that the basic reproduction number associated with $(h_1,h_2)$ is given as

3.21 $$\begin{aligned} R_0(h_1,h_2):=r(K^{h_1,h_2}). \end{aligned}$$

Especially, if $h_1=h_2=S_2$, then the basic reproduction number associated with $E_2$ for virus transmission is denoted as

3.22 $$\begin{aligned} R_0:=R_0(S_2,S_2)=r(K^{S_2,S_2}). \end{aligned}$$

Since the uniqueness of $E_2$ is not known, $R_0$ defined in (3.22) depends on $E_2$, and the definition given in (3.21) allows a more general basic reproduction number which will be used in the following.

Let ${\bar{I}}=e^{\lambda t}\varphi$ and $V=e^{\lambda t}\psi$. Then $(\lambda ,\varphi ,\psi )$ satisfies an eigenvalue problem

3.23 $$\begin{aligned} \begin{aligned}&\lambda \varphi =d_p\varphi _{xx}+\omega \varphi _x+b\beta e^{-(\omega /d_p)x}h_1\psi -\delta \varphi ,~x\in (0,x_l),\\&\lambda \psi =d_v\psi _{xx}+q\delta e^{(\omega /d_p)x}\varphi -\mu _v\psi -\beta h_2\psi ,~x\in (0,x_l),\\&\varphi '(0)=\varphi '(x_l)=\psi '(0)={\psi '(x_l)}=0. \end{aligned} \end{aligned}$$

Note that (3.23) is a cooperative system. By the Krein-Rutman theorem, (3.23) has a unique principal eigenvalue $\lambda _0(h_1,h_2)$ with a strongly positive eigenfunction $({\hat{\varphi }},{\hat{\psi }})$. Applying the similar arguments of Theorem 3.1 (i) in Wang and Zhao (2012), one can obtain the following conclusion.

#### Lemma 3.7

$\lambda _0(h_1,h_2)$ and $R_0(h_1,h_2)-1$ have the same sign.

To apply the uniform persistence theory in Magal and Zhao (2005); Smith and Zhao (2001); Zhao (2017), we consider the susceptible phytoplankton-nutrient model (3.16) (the sub-system of (2.1)–(2.3) with $I=V=0$).

Let

$$\begin{aligned}&{\mathcal {U}}:=\{(S,N)\in C([0,x_l],{\mathbb {R}}^2):S(x)\ge 0,~N(x)\ge 0~\text {on}~[0,x_l]\},\\&{\mathcal {U}}^*:=\{(S,N)\in {\mathcal {U}}:S(\cdot )\not \equiv 0\}. \end{aligned}$$

Denote the solution semiflow $\Sigma (t):{\mathcal {U}}\rightarrow {\mathcal {U}}$ of (3.16) as

$$\begin{aligned} \Sigma (t)(u_0)(x)=(S(x,t,u_0),N(x,t,u_0)),~x\in [0,x_l],~t\ge 0, \end{aligned}$$

where $(S(\cdot ,t,u_0),N(\cdot ,t,u_0))$ is the solution of (3.16) with the initial value $u_0=(S_0,N_0)\in {\mathcal {U}}$. Following Lemmas 3.7 and 3.8 in Yan et al. (2022), one can obtain the following conclusion.

#### Lemma 3.8

If $R_p>1$, then (3.16) has a global attractor $\Delta _0$ in ${\mathcal {U}}^*$ satisfying $\Sigma (t)(\Delta _0)=\Delta _0$ and $(S_2,N_2)\in \Delta _0\subset {{\,\textrm{Int}\,}}(C([0,x_l],{\mathbb {R}}^2_+)$.

From Theorem 3.3, we have $\lim \limits _{t\rightarrow \infty }(S(\cdot ,t),N(\cdot ,t))= (0,N^0)$ when $R_p<1$. The numerical simulations in the next section suggest that the attractor $\Delta _0$ when $R_p>1$ only contains the steady state $(S_2,N_2)$.

Let

3.24 $$\begin{aligned}{} & {} {\mathcal {W}}^*:=\{(S,I,V,N)\in {\mathcal {W}}:S(\cdot )\not \equiv 0,I(\cdot )\not \equiv 0~ \text {and}~V(\cdot )\not \equiv 0\}\nonumber \\{} & {} ~\text {and}~\partial {\mathcal {W}}^*:={\mathcal {W}}\setminus {\mathcal {W}}^*. \end{aligned}$$

Here ${\mathcal {W}}$ is defined in (3.1). For any $(S,N)\in {\mathcal {U}}$, we introduce a projection ${\mathcal {H}}$ by ${\mathcal {H}}(S,N)=S$. Let

3.25 $$\begin{aligned} \Lambda _0={\mathcal {H}}(\Delta _0),~S_*(x)=\inf \limits _{S\in \Lambda _0}S(x),~S^*(x)=\sup \limits _{S\in \Lambda _0}S(x) ~\text {for any}~x\in [0,x_l].\nonumber \\ \end{aligned}$$

The uniform persistence shown in Lemma 3.8 implies that $0<S_*(x)\le S^*(x)$ for $x\in [0,x_l]$ Substituting $h_1=S_*$, $h_2=S^*$ in (3.20) and (3.23) respectively, we can define the principal eigenvalue $\lambda _0(S_*,S^*)$ and the basic reproduction number $R_0(S_*,S^*)$ associated with $(S_*,S^*)$. It follows from Lemma 3.7 that $\lambda _0(S_*,S^*)$ and $R_0(S_*,S^*)-1$ have the same sign.

Let $\Upsilon _0:=\{(S,0,0,N)\in {\mathcal {W}}:(S,N)\in \Delta _0\}$. It will prove that $E_1$ and $\Upsilon _0$ are uniform weak repellers with respect to ${\mathcal {W}}^*$.

#### Lemma 3.9

If $R_p>1$, then $E_1$ is a uniform weak repeller for (2.1)–(2.3) with respect to ${\mathcal {W}}^*$, that is, there is a $\upsilon _1>0$ satisfying $\limsup \limits _{t\rightarrow \infty }{{\,\textrm{dist}\,}}(\Theta (t)\sigma _0,E_1)\ge \upsilon _1$ for any $\sigma _0=(S_0,I_0,V_0,N_0)\in {\mathcal {W}}^*$.

#### Proof

If $R_p>1$, there is an $\epsilon >0$ satisfying $R_p^{\epsilon }=\mu _p^*/(\mu _p+\epsilon )>1$. Note that both *f* and *g* are continuous. Thus, for the above $\epsilon >0$, there is a $\upsilon _1>0$ such that

3.26 $$\begin{aligned} {rg(N)f(L(x,S+\theta I))}-\eta (S+\theta I)S-b\beta SV>rg(N^0)f(L(x,0))-\epsilon \nonumber \\ \end{aligned}$$

when $\Vert (S,I,V,N)-(0,0,0,N^0)\Vert <\upsilon _1$ on $[0,x_l]$. By (3.9), $\mu _p^*-\mu _p-\epsilon >0$ is the principal eigenvalue of

$$\begin{aligned}&\lambda \xi =d_p\xi ''-\omega \xi '+(rg(N^0)f(L(x,0))-\mu _p-\epsilon )\xi ,~x\in (0,x_l),\\&d_p\xi '(0)-\omega \xi (0)=d_p\xi '(x_l)-\omega \xi (x_l)=0 \end{aligned}$$

with the positive eigenvalue function $\xi ^{\epsilon }(x)$.

Assume that the conclusion is not true. Then we can find a $\sigma _0\in {\mathcal {W}}^*$ satisfying

3.27 $$\begin{aligned} \limsup \limits _{t\rightarrow \infty }\Vert \Theta (t)\sigma _0-(0,0,0,N^0)\Vert <\upsilon _1. \end{aligned}$$

This suggests that

$$\begin{aligned} \Vert (S(\cdot ,t,\sigma _0),I(\cdot ,t,\sigma _0),V(\cdot ,t,\sigma _0),N(\cdot ,t,\sigma _0))-(0,0,0,N^0)\Vert <\upsilon _1,~t\ge T_1 \end{aligned}$$

for some sufficiently large $T_1$. It follows from the strong maximum principle and the Hopf boundary lemma that $S(\cdot ,T_1,\sigma _0)>0$ since $\sigma _0\in {\mathcal {W}}^*$. Hence, there exists a $c_1>0$ such that $S(\cdot ,T_1,\sigma _0)\ge c_1\xi ^{\epsilon }(\cdot )$. Let ${\hat{S}} =Se^{-(\omega /d_p)x}$ and ${\hat{\xi }}^{\epsilon } =\xi ^{\epsilon } e^{-(\omega /d_p)x}$. From (3.26) and the *S*-equation in (2.1), we have

$$\begin{aligned}&{\hat{S}}_t\ge d_p{\hat{S}}_{xx}+\omega {\hat{S}}_x+(rg(N^0)f(L(x,0)) -\mu _p-\epsilon ){\hat{S}},~x\in (0,x_l),~t>T_1,\\&{\hat{S}}_x(0,t)={\hat{S}}_x(x_l,t)=0,~t\ge T_1,\\&{\hat{S}}(x,T_1,\sigma _0)\ge c_1{\hat{\xi }}^{\epsilon }(x),~x\in [0,x_l]. \end{aligned}$$

It is easy to see that $c_1e^{(\mu _p^*-\mu _p-\epsilon )(t-T_1)}{\hat{\xi }}^{\epsilon }(x)$ is a solution of

$$\begin{aligned}&{\hat{S}}_t=d_p{\hat{S}}_{xx}+\omega {\hat{S}}_x+(rg(N^0)f(L(x,0)) -\mu _p-\epsilon ){\hat{S}},~x\in (0,x_l),~t>T_1,\\&{\hat{S}}_x(0,t)={\hat{S}}_x(x_l,t)=0,~t\ge T_1,\\&{\hat{S}}(x,T_1)=c_1{\hat{\xi }}^{\epsilon }(x),~x\in [0,x_l]. \end{aligned}$$

From the comparison theorem of parabolic system, we obtain

$$\begin{aligned}{\hat{S}}(\cdot ,t,\sigma _0)\ge c_1e^{(\mu _p^*-\mu _p-\epsilon )(t-T_1)}{\hat{\xi }}^{\epsilon }(\cdot )\end{aligned}$$

for any $t\ge T_1$. Then $\lim \limits _{t\rightarrow \infty }S(\cdot ,t,\sigma _0)=\lim \limits _{t\rightarrow \infty }{\hat{S}}(\cdot ,t,\sigma _0)e^{(\omega /d_p)x}=\infty$ since $R_p^{\epsilon }>1$. It contradicts (3.27). Therefore, $E_1$ is a uniform weak repeller with respect to ${\mathcal {W}}^*$. $\square$

#### Lemma 3.10

Let $S_*$ and $S^*$ be defined as in (3.25). If $R_p>1$ and $R_0(S_*,S^*)>1$, then $\Upsilon _0$ is a uniform weak repeller for (2.1)–(2.3) with respect to ${\mathcal {W}}^*$, that is, there is a $\upsilon _2>0$ satisfying $\limsup \limits _{t\rightarrow \infty }{{\,\textrm{dist}\,}}(\Theta (t)\sigma _0,\Upsilon _0)\ge \upsilon _2$ for any $\sigma _0=(S_0,I_0,V_0,N_0)\in {\mathcal {W}}^*$.

#### Proof

If $R_0(S_*,S^*)>1$, then $\lambda _0(S_*,S^*)>0$. This indicates that there exists a $\upsilon _2>0$ such that $\lambda _0(S_*-\upsilon _2,S^*+\upsilon _2)>0$ is the principal eigenvalue of

$$\begin{aligned} \begin{aligned}&\lambda \varphi =d_p\varphi _{xx}+\omega \varphi _x+b\beta e^{-(\omega /d_p)x}(S_*-\upsilon _2)\psi -\delta \varphi ,~x\in (0,x_l),~t>0,\\&\lambda \psi =d_v\psi _{xx}+q\delta e^{(\omega /d_p)x}\varphi -\mu _v\psi -\beta (S^*+\upsilon _2)\psi ,~x\in (0,x_l),~t>0,\\&\varphi _x(0,t)=\varphi _x(x_l,t)=\psi _x(0,t)=\psi _x(x_l,t)=0,~t>0 \end{aligned} \end{aligned}$$

with the positive eigenvalue function $({\hat{\varphi }}^{\upsilon _2},{\hat{\psi }}^{\upsilon _2})$.

If the conclusion does not hold, then for the above $\upsilon _2$, there exists a $\sigma _0\in {\mathcal {W}}^*$ satisfying $\limsup \nolimits _{t\rightarrow \infty }{{\,\textrm{dist}\,}}(\Theta (t)\sigma _0,\Upsilon _0)<\upsilon _2$. This means that

3.28 $$\begin{aligned}{} & {} \limsup \limits _{t\rightarrow \infty }{{\,\textrm{dist}\,}}(S(\cdot ,t,\sigma _0),\Lambda _0)<\upsilon _2, ~\limsup \limits _{t\rightarrow \infty }\Vert I(\cdot ,t,\sigma _0)\Vert<\upsilon _2,\nonumber \\{} & {} \limsup \limits _{t\rightarrow \infty }\Vert V(\cdot ,t,\sigma _0)\Vert <\upsilon _2 \end{aligned}$$

and then there is a $T_2>0$ such that

$$\begin{aligned} {{\,\textrm{dist}\,}}(S(\cdot ,t,\sigma _0),\Lambda _0)<\upsilon _2~\text {for all}~t\ge T_2. \end{aligned}$$

Since $\Lambda _0$ is compact, we can find an ${\bar{S}}^t\in \Lambda _0$ satisfying

$$\begin{aligned} \Vert S(\cdot ,t,\sigma _0)-{\bar{S}}^t(\cdot )\Vert <\upsilon _2~\text {for all}~t\ge T_2. \end{aligned}$$

Hence,

$$\begin{aligned} S_*(\cdot )-\upsilon _2\le {\bar{S}}^t(\cdot )-\upsilon _2<S(\cdot ,t,\sigma _0)<\bar{S}^t(\cdot )+\upsilon _2\le S^*(\cdot )+\upsilon _2~\text {for all}~t\ge T_2. \end{aligned}$$

From the *I*- and *V*-equations in (2.1), we let $\bar{I}=Ie^{-(\omega /d_p)x}$ and have

$$\begin{aligned} \begin{aligned}&{\bar{I}}_t\ge d_p{\bar{I}}_{xx}+\omega {\bar{I}}_x+b\beta e^{-(\omega /d_p)x}(S_*-\upsilon _2)V -\delta {\bar{I}},~x\in (0,x_l),~t>T_2,\\&V_t\ge d_vV_{xx}+q\delta e^{(\omega /d_p)x}{\bar{I}}-\mu _vV-\beta (S^*+\upsilon _2)V,~x\in (0,x_l),~t>T_2,\\&{\bar{I}}_x(0,t)={\bar{I}}_x(x_l,t)=V_x(0,t)=V_x(x_l,t)=0,~t>T_2. \end{aligned} \end{aligned}$$

By the strong maximum principle and the Hopf boundary lemma, $\bar{I}(x,T_2,\sigma _0)>0$ and $V(x,T_2,\sigma _0)>0$ for $\sigma _0\in {\mathcal {W}}^*$. There is a $c_2>0$ satisfying $({\bar{I}}(x,T_2,\sigma _0),V(x,T_2,\sigma _0))\ge c_2({\hat{\varphi }}^{\upsilon _2},{\hat{\psi }}^{\upsilon _2})$ on $x\in [0,x_l]$. It can be seen that $c_2e^{\lambda _0(S_*-\upsilon _2,S^*+\upsilon _2)(t-T_2)}({\hat{\varphi }}^{\upsilon _2},{\hat{\psi }}^{\upsilon _2})$ is a solution of

$$\begin{aligned} \begin{aligned}&{\bar{I}}_t= d_p{\bar{I}}_{xx}+\omega {\bar{I}}_x+b\beta e^{-(\omega /d_p)x}(S_*-\upsilon _2)V -\delta {\bar{I}},~x\in (0,x_l),~t>T_2,\\&V_t=d_vV_{xx}+q\delta e^{(\omega /d_p)x}{\bar{I}}-\mu _vV-\beta (S^*+\upsilon _2)V,~x\in (0,x_l),~t>T_2,\\&{\bar{I}}_x(0,t)={\bar{I}}_x(x_l,t)=V_x(0,t)=V_x(x_l,t)=0,~t>T_2. \end{aligned} \end{aligned}$$

Applying the comparison theorem of parabolic system again, we have

$$\begin{aligned}{} & {} ({\bar{I}}(x,t,\sigma _0),V(x,t,\sigma _0))\ge c_2e^{\lambda _0(S_*-\upsilon _2,S^*+\upsilon _2)(t-T_2)}({\hat{\varphi }}^{\upsilon _2},\\{} & {} {\hat{\psi }}^{\upsilon _2})~\text {for all}~x\in [0,x_l],~t\ge T_2. \end{aligned}$$

This implies that ${\bar{I}}(x,t,\sigma _0),V(x,t,\sigma _0)$ are unbounded since $\lambda _0(S_*-\upsilon _2,S^*+\upsilon _2)>0$. This contradicts with (3.28). The proof is completed. $\square$

Now we are ready to prove the existence of $E_3$ and uniform persistence of model (2.1)–(2.3) when $R_p>1$ and $R_0(S_*,S^*)>1$.

#### Theorem 3.11

Let $S_*$ and $S^*$ be defined as in (3.25). If $R_p>1$ and $R_0(S_*,S^*)>1$, then model (2.1)–(2.3) is uniformly persistent for $({\mathcal {W}}^*,\partial {\mathcal {W}}^*)$, that is, there exists a $\upsilon >0$ satisfying

3.29 $$\begin{aligned} \liminf _{t\rightarrow \infty }S(\cdot ,t,\sigma _0)>\upsilon ,~\liminf _{t\rightarrow \infty }I(\cdot ,t,\sigma _0)>\upsilon ,~ \liminf _{t\rightarrow \infty }V(\cdot ,t,\sigma _0)>\upsilon \end{aligned}$$

for any $\sigma _0=(S_0,I_0,V_0,N_0)\in {\mathcal {W}}^*$. Moreover, model (2.1)–(2.3) possesses at least one endemic steady state $E_3$.

#### Proof

Applying the Hopf boundary lemma and strong maximum principle again, one can obtain

3.30 $$\begin{aligned} S(\cdot ,t,\sigma _0)>0,~I(\cdot ,t,\sigma _0)>0,~V(\cdot ,t,\sigma _0)>0,~N(\cdot ,t,\sigma _0)>0 \end{aligned}$$

for any $t>0$ and $\sigma _0\in {\mathcal {W}}^*$, where ${\mathcal {W}}^*$ is defined in (3.24). Thus, ${\mathcal {W}}^*$ is positively invariant under $\Theta (t)$. Set $M_\partial :=\{\sigma _0\in \partial {\mathcal {W}}^*:\Theta (t)\sigma _0\in \partial {\mathcal {W}}^*~\text {for any}~t\ge 0\}$ and let $\omega (\sigma _0)$ be the omega limit set of the forward orbit $O^+(\sigma _0):=\{\Theta (t)\sigma _0:t\ge 0\}$.

We claim that $\omega (\sigma _0)\subset E_1\cup \Upsilon _0$ for any $\sigma _0\in M_\partial$. Note that $\Theta (t)\sigma _0\in M_\partial$ for any fixed $\sigma _0\in M_\partial$. This implies that $S(\cdot ,t,\sigma _0)\equiv 0$ or $I(\cdot ,t,\sigma _0)\equiv 0$ or $V(\cdot ,t,\sigma _0)\equiv 0$ for $\sigma _0\in M_\partial$. The *I* and *V* equations in (2.1) are a cooperative system. Hence, $I(\cdot ,t,\sigma _0)\equiv 0$ if and only if $V(\cdot ,t,\sigma _0)\equiv 0$. This means that we only need to investigate the following three cases:

(i) if $S_0\equiv 0, V_0\equiv 0$, then $S(\cdot ,t,\sigma _0)\equiv 0$ for any $t\ge 0$ from the uniqueness of solutions. The *I*-equation in model (2.1) reduces to

$$\begin{aligned}&I_t=d_pI_{xx}-\omega I_x-\delta I,~x\in (0,x_l),~t>0,\\&d_pI_x(0,t)-\omega I(0,t)=d_pI_x(x_l,t)-\omega I(x_l,t)=0,~t>0. \end{aligned}$$

Similar to the derivation process in (3.13)–(3.15), we have

$$\begin{aligned}\limsup \limits _{t\rightarrow \infty }I(\cdot ,t,\sigma _0)=0,~ \limsup \limits _{t\rightarrow \infty }V(\cdot ,t,\sigma _0)=0~\text{ and }~ \limsup \limits _{t\rightarrow \infty }N(\cdot ,t,\sigma _0)=N_0.\end{aligned}$$

Thus, $$\omega (\sigma _0)=E_1$$.

(ii) if $S_0\equiv 0, V_0\ne 0$, then $S(\cdot ,t,\sigma _0)\equiv 0$. Similar to case (i), we have $\omega (\sigma _0)=E_1$.

(iii) if $S_0\ne 0, V_0\equiv 0$, then $\limsup \limits _{t\rightarrow \infty }I(\cdot ,t,\sigma _0)=0$ and $\limsup \limits _{t\rightarrow \infty }V(\cdot ,t,\sigma _0)=0$ from (3.13) and (3.14). By the theory of asymptotic autonomous systems (see Theorem 1.8 in Mischaikow et al. 1995 or Theorem 4.1 in Thieme 1992), (2.1) reduces to (3.16). From Lemma 3.8, $(S(\cdot ,t,u_0),N(\cdot ,t,u_0))$ will eventually enter $\Delta _0$. Hence $\omega (\sigma _0)\subset \Upsilon _0$.

By Lemmas 3.9 and 3.10, $E_1$ and $\Upsilon _0$ are uniform weak repellers for ${\mathcal {W}}^*$. To obtain our conclusion, we let $\varpi :{\mathcal {W}}\rightarrow [0,\infty )$ satisfying

$$\begin{aligned}{} & {} \varpi (\sigma _0):=\min \left\{ \min \limits _{x\in [0,x_l]}S_0(x),\min \limits _{x\in [0,x_l]}I_0(x),\min \limits _{x\in [0,x_l]}V_0(x)\right\} \\{} & {} ~\text {for any}~\sigma _0=(S_0,I_0,V_0,N_0)\in {\mathcal {W}}. \end{aligned}$$

By (3.30), we get $\varpi ^{-1}(0,\infty )\subseteq {\mathcal {W}}^*$ and $\varpi (\Theta (t)\sigma _0)>0$ for any $t>0$ if $\varpi (\sigma _0)>0$ or $\sigma _0\in {\mathcal {W}}^*$ with $\varpi (\sigma _0)=0$. For the semiflow $\Theta (t):{\mathcal {W}}\rightarrow {\mathcal {W}}$, $\varpi$ is a generalized distance function. In view of the above analysis, we conclude that $\omega (\sigma _0)\subset E_1\cup \Upsilon _0$ for any $\sigma _0\in \ M_\partial$, and there is no cycle in $M_\partial$ from $E_1\cup \Upsilon _0$ to $E_1\cup \Upsilon _0$. Furthermore, $E_1$ and $\Upsilon _0$ are isolated in ${\mathcal {W}}$. Let $W^s(E_1)$ and $W^s(\Upsilon _0)$ denote the stable sets of $E_1$ and $\Upsilon _0$ respectively. It is easy to see that $W^s(E_1)\cap {\mathcal {W}}^*=\emptyset$ and $W^s(\Upsilon _0)\cap {\mathcal {W}}^*=\emptyset$. From Remark 3.2, $\Theta (t)$ has a global attractor in ${\mathcal {W}}$. By Theorem 3 in Smith and Zhao (2001), there exists a $\upsilon >0$ such that $\min \limits _{\phi \in \omega (\sigma _0)}\varpi (\phi )>\upsilon$ for any $\sigma _0\in {\mathcal {W}}^*$. It follows that (3.29) holds and the uniform persistence is valid. Applying Theorem 3.7 and Remark 3.10 in Magal and Zhao (2005), $\Theta (t):{\mathcal {W}}^*\rightarrow {\mathcal {W}}^*$ admits a global attractor. By Theorem 4.7 in Magal and Zhao (2005), model (2.1)–(2.3) has at least one endemic steady state $E_3\in {\mathcal {W}}^*$. Similar to the proof in Lemma 3.5, we have $S_3(x)>0,N_3(x)>0$ on $[0,x_l]$. Let $\bar{I}_3=I_3e^{-(\omega /d_p)x}$. From the second and third equalities in (3.7), we obtain

$$\begin{aligned} -d_p{\bar{I}}_3''-\omega {\bar{I}}_3'+\delta {\bar{I}}_3=b\beta S_3V_3e^{-(\omega /d_p)x}\ge 0,~x\in (0,x_l),~ {\bar{I}}_3(0)=\bar{I}_3(x_l)=0 \end{aligned}$$

and

$$\begin{aligned} -d_vV_3''(x)+(\mu _v+\beta S_3)V_3=q\delta I_3\ge 0,~x\in (0,x_l),~V'_3(0)=V'_3(x_l)=0. \end{aligned}$$

Thus, $I_3(x)>0$ and $V_3(x)>0$ on $[0,x_l]$ following the strong maximum principle. The proof is complete. $\square$

**Fig. 2:**
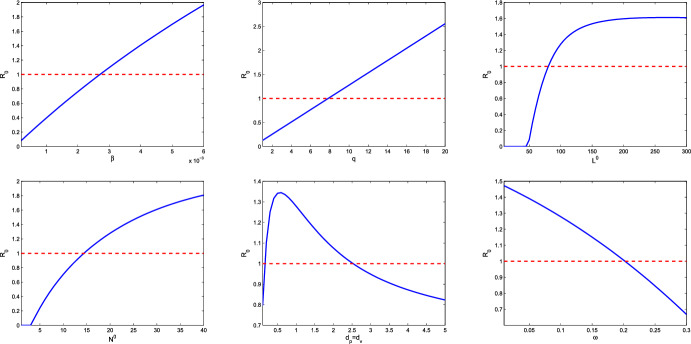
Dependence of $R_0$ on some model parameters. Here $L^0=100, N^0=20$ and the remaining parameter values are derived from Table 1

From numerical simulations, we speculate that the global attractor $\Delta _0=\{(S_2,N_2)\}$ in Lemma 3.8 and the basic reproduction number $R_0(S_*,S^*)=R_0(S_2,S_2)=R_0$ can be uniquely determined. Theorem 3.11 shows that lytic viruses will be transmitted in the phytoplankton population if $R_p>1$ and $R_0>1$. Hence $R_0=1$ is a threshold for lytic viruses from persistence to extinction. Figure 2 reveals the evolution of $R_0$ with respect to model parameters. From numerical simulations in Sect. 3.3, the detailed lytic virus transmission is more complicated. The endemic state may be a positive steady state $E_3$, or a positive spatially inhomogeneous periodic solution.

### Simulations

**Fig. 3:**
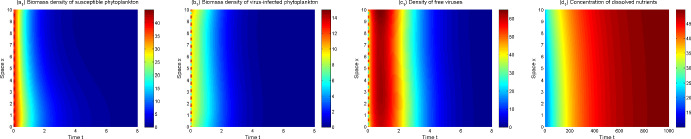
The solutions of model (2.1)–(2.3) converge to $E_1$ for $\mu _p=1,\mu _v=1.2$

**Fig. 4:**
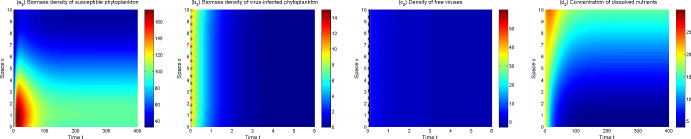
The solutions of model (2.1)–(2.3) converge to $E_2$ for $\mu _p=0.1,\mu _v=24$

**Fig. 5:**
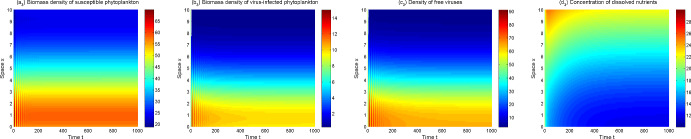
The solutions of model (2.1)–(2.3) converge to $E_3$ for $\mu _p=0.1,\mu _v=1.6$

**Fig. 6:**
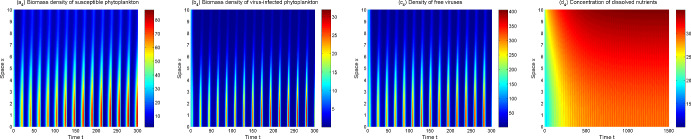
The solutions of model (2.1)–(2.3) converge to a positive spatially inhomogeneous periodic solution for $\mu _p=0.1,\mu _v=0.6$

In view of the above model analysis, we do some numerical simulations to further describe dynamics of the model (2.1)–(2.3). From Figs. 3, 4, 5 and 6, one can observe that the solutions of model (2.1)–(2.3) converge to different asymptotic states for different $\mu _p,\mu _v$. Here ecologically reasonable parameter values are from Table 1 and the initial conditions are $S_0(x)=40+5\sin x, I_0(x)=10+5\cos x, V_0(x)=40+20\cos x, N_0(x)=20+10\sin x$ on [0, 10].

In Fig. 3, the extinct steady state $E_1$ is the attractor with $R_p=0.49$. Phytoplankton are extinct and thus the lytic virus does not spread in the water column. Nutrients tend to the input concentration $N^0$ and are spatially uniformly distributed. Figure 4 shows that the (possibly unique) disease-free steady state $E_2$ is the attractor when $R_p=4.91$ and $R_0=0.13$. Phytoplankton invade aquatic ecosystems and exhibit vertical aggregation. Lytic virus still cannot be transmitted among phytoplankton because lytic virus mortality is high.

The model (2.1)–(2.3) is uniformly persistent and an endemic steady state $E_3$ appears to be the asymptotic state when $R_p=4.91$ and $R_0=1.59$ (see Fig. 5). As $\mu _v$ decreases further ($R_p=4.91$, $R_0=3.24$), a positive spatially inhomogeneous periodic solution becomes the asymptotic state, which arises from bifurcate from $E_3$ via a Hopf bifurcation (see Fig. 6). Both Figs. 5 and 6 show the lytic virus is prevalent in the phytoplankton, either in a form of spatially heterogenous steady state (Fig. 5) or a spatially heterogenous temporal-oscillatory fashion (Fig. 6). It is noticeable that susceptible phytoplankton, virus-infected phytoplankton and lytic viruses aggregate near the water surface (for only short time in the oscillatory case). Comparing Fig. 4 (a_2_)–(b_2_) and Fig. 5 (a_3_)–(b_3_), one can also observe that lytic viruses reduce phytoplankton biomass.

## Phytoplankton blooms and lytic virus transmission

Phytoplankton blooms are an important manifestation of the pollution of the aquatic environments, and even lead to the collapse of entire aquatic ecosystems. It has been shown that the wide spreading of lytic viruses transmission among phytoplankton can control phytoplankton blooms from observations and experiments (Fuhrman 1999; Kuhlisch et al. 2021). In the following discussion, we will verify these observations and experimental results with the proposed model (2.1)–(2.3). It is noted that the role of ecological factors in the interaction of phytoplankton blooms and lytic virus transmission is not very clear. It is also necessary and significant to explore the effects of ecological factors in this process.

We focus on the environmental parameters related to viral transmission and ecological factors in model (2.1)–(2.3). Those parameters include viral infection-related rates $\beta ,q$, spatial ecological factors $d_p,d_v,d_n,\omega$, and resource-related ecological factors $L^0$ and $N^0$. In the figures below, we show the evolution of asymptotic states (steady state solutions $E_1,E_2,E_3$ and the spatially inhomogeneous periodic solution) for different parameter values. When the solutions of (2.1)–(2.3) converge to one of $E_1,E_2,E_3$ or the spatially inhomogeneous periodic solution, numerical bifurcation diagrams show the evolution trend of densities of susceptible phytoplankton ($(1/x_l)\int _0^{x_l}S(x)dx$), virus-infected phytoplankton ($(1/x_l)\int _0^{x_l}I(x)dx$), and free lytic viruses ($(1/x_l)\int _0^{x_l}V(x)dx$). For the time-periodic solutions, the minimum and maximum values are shown. Time series diagrams reveal the evolution of densities of susceptible phytoplankton (($(1/x_l)\int _0^{x_l}S(x,t)dx$), infected phytoplankton ($(1/x_l)\int _0^{x_l}I(x,t)dx$) and lytic viruses ($(1/x_l)\int _0^{x_l}V(x,t)dx$) over time. The parameter values of the numerical analysis used here are derived from Table 1. The simulations are implemented in MATLAB via the finite difference method.

**Fig. 7:**
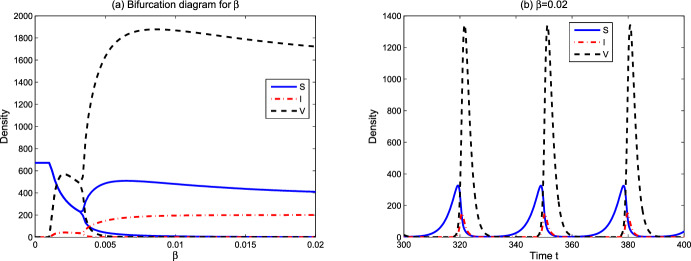
**a** Effect of the transmission coefficient $\beta$ on the density of susceptible phytoplankton, virus-infected phytoplankton and lytic viruses. **b** Time series of density of phytoplankton and viruses

We first examine the effect of lytic virus transmission parameters $\beta ,q$. Changes in these parameters are closely related to ecological factors such as temperature, salinity (Demory et al. 2021). The transmission coefficient $\beta$ is an important indicator for assessing host resistance to infection. Figure 7 displays the variation of phytoplankton and lytic virus densities in model (2.1)–(2.3) with the transmission coefficient $\beta$. For $\beta =0$, one can observe that lytic viruses do not spread among phytoplankton and that susceptible phytoplankton biomass is at a high level. When $\beta$ increases, susceptible phytoplankton biomass gradually declines, while lytic virus loads ascend sharply and a significant proportion of phytoplankton are infected by viruses. In Fig. 7a, b, spatially inhomogeneous periodic solutions bifurcate from positive steady states through a Hopf bifurcation at $\beta =0.0032$. This shows that increasing the transmission coefficient will lead to persistent phytoplankton blooms, but a large transmission coefficient will cause a large amplitude pulse bloom which occurs periodically about every thirty days. In the latter case, an increase of susceptible phytoplankton precedes the peak of virus transmission, then the phytoplankton population is at a low level for a long time until the next wave.

**Fig. 8:**
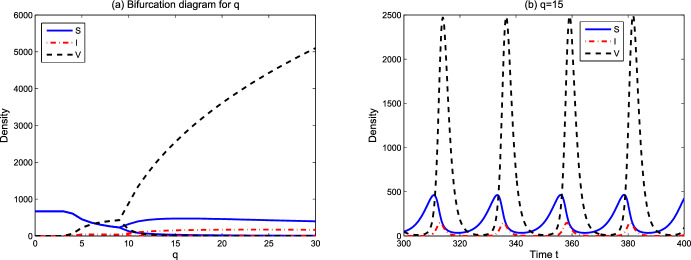
**a** Effect of the burst size *q* on the density of susceptible phytoplankton, virus-infected phytoplankton and lytic viruses. **b** Time series of density of phytoplankton and viruses

The lytic virus burst size varies with host genotype or environmental conditions. It is an important factor for describing the transmission of lytic viruses among phytoplankton (Edwards and Steward 2018). Figure 8 shows an evolution of asymptotic states in model (2.1)–(2.3) for varying burst size *q* similar to the one for transmission coefficient. The dynamics of the model progress from a disease-free steady state, to an endemic steady state, and then to a spatially inhomogeneous periodic solution. This indicates that the small burst size does not cause phytoplankton infection and that only a certain degree of burst size can lead to the spread of lytic viruses among phytoplankton and a decrease in total phytoplankton biomass.

*Emiliana huxleyi* are eukaryotic microscopic phytoplankton that are widely distributed in oceans or brackish waters. They are one of the important producers in marine ecosystems, especially playing an important role in the carbon cycle of oceans. E*miliana huxleyi* have frequent, large-scale blooms each year. It is known that *Emiliana huxleyi* can be massively infected by a lytic virus with a large double-stranded DNA structure. The lytic virus is called *Emiliana huxleyi* virus and causes massive mortality of *Emiliana huxleyi*. In Fuhrman (1999); Kuhlisch et al. (2021), the authors stated that *Emiliana huxleyi* blooms are often terminated by *Emiliana huxleyi* virus from experiments and observations.

The periodic pattern shown in Fig. 7b is consistent with the observations and experimental results of *Emiliana huxleyi*-lytic virus interactions in Fuhrman (1999); Kuhlisch et al. (2021). At the beginning of a bloom cycle, both phytoplankton and lytic virus densities are at a very low value. After that, the susceptible phytoplankton biomass rises rapidly and reaches a maximum, while the virus density remains almost constant. Phytoplankton blooms occur at this time. As the time progresses further, there is a sudden and dramatic increase in lytic virus density. This indicates a large-scale spread of lytic viruses among phytoplankton. During this process, the total phytoplankton biomass declines sharply. When the virus reaches its maximum, phytoplankton biomass tends to almost zero, thus the bloom ends. In the last stage of this bloom cycle, the viral density decreases, and is again at a low value together with phytoplankton. Figure 8b also exhibits a similar periodic bloom cycle pattern. The above findings effectively validate the large-scale transmission of lytic viruses to terminate phytoplankton blooms.

**Fig. 9:**
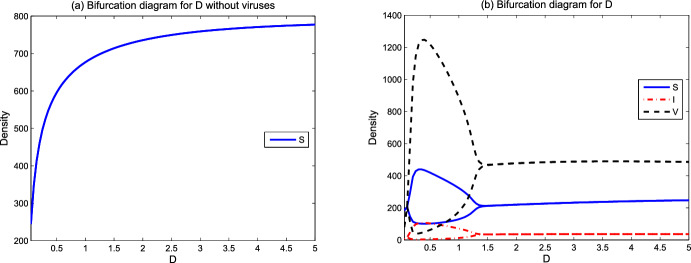
Effect of the vertical turbulent diffusivity $D=d_p=d_v=d_n$ on the density of susceptible phytoplankton, virus-infected phytoplankton and lytic viruses

**Fig. 10:**
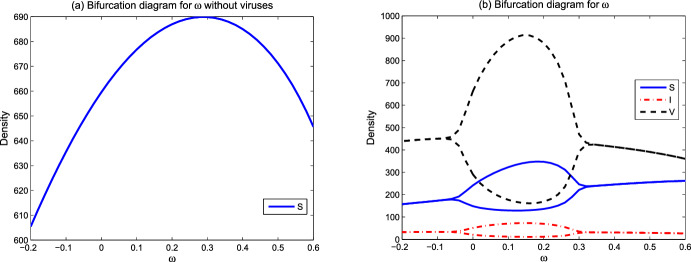
Effect of the sinking or buoyant velocity $\omega$ on the density of susceptible phytoplankton, virus-infected phytoplankton and lytic viruses

The evolution of phytoplankton and lytic virus densities with different spatial ecological factors (turbulent diffusivity and directional movement velocity) can be seen in Figs. 9 and 10. Phytoplankton, viruses, and nutrients all move randomly in the water column with turbulence, so it is assumed that they have the same diffusion coefficient $D=d_p=d_v=d_n$. If there are no viruses in the aquatic environment, the phytoplankton biomass rises rapidly and reaches a high value with increasing turbulence intensity. It is because adequate nutrient transport in the water column facilitates better phytoplankton growth (see Fig. 9a). When the lytic virus spreads among phytoplankton, the biomass of phytoplankton, including susceptible and infected phytoplankton, remains almost unchanged for $D\in (0.02,5)$ except the periodic oscillatory patterns occurring for the intermediate diffusion rate (see Fig. 9b). In this process, there are two stability switches from steady states to periodic oscillations, and then back to steady states. This illustrates that the presence of lytic viruses not only leads to complex dynamics, but also effectively reduces the phytoplankton biomass. Comparing Fig. 10a, b, similar conclusions are obtained for the directional movement velocity $\omega$. This once again confirms that lytic virus transmission can effectively control phytoplankton blooms.

**Fig. 11:**
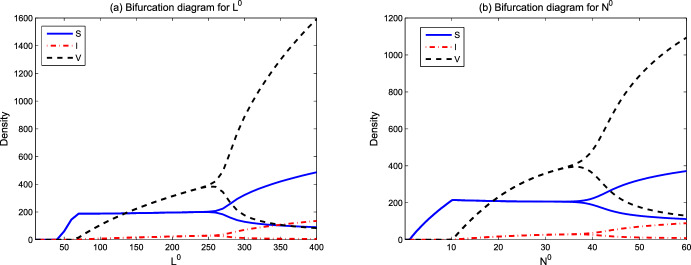
Effects of the water surface light intensity $L^0$ and sediment input nutrient concentration $N^0$ on the density of susceptible phytoplankton, virus-infected phytoplankton and lytic viruses

**Fig. 12:**
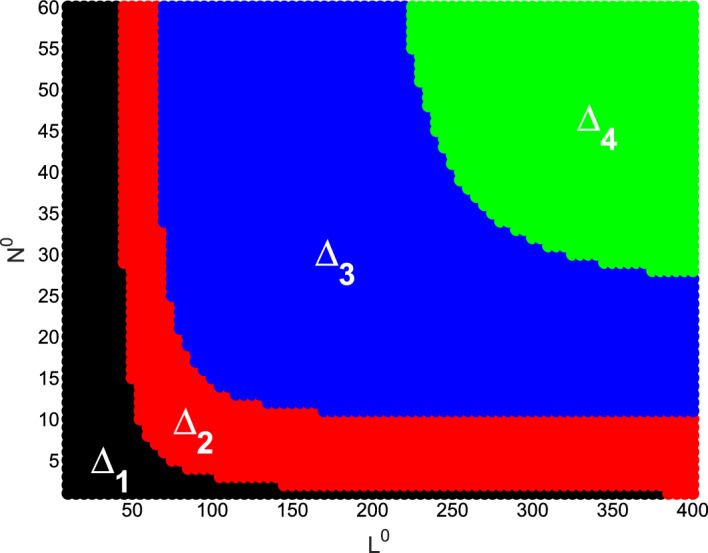
Parameter regions of $L^0$ versus $N^0$ for the survival and extinction of phytoplankton and lytic viruses. Phytoplankton are extinct in $\Delta _1$ ($R_p<1$), phytoplankton survive while lytic viruses become extinct in $\Delta _2$ ($R_p>1,R_0<1$), lytic viruses spread among phytoplankton in a steady state form in $\Delta _3$ ($R_p>1,R_0>1$) or as a spatially inhomogeneous periodic solution form in $\Delta _4$ ($R_p>1,R_0>1$)

Light and nutrients are two important ecological factors, and their abundance directly affects the invasion of phytoplankton and the spread of lytic viruses. We choose the water surface light intensity $L^0$ and sediment nutrient input concentration $N^0$ as typical resource-related ecological factors to explore the survival and extinction of phytoplankton and lytic viruses. Figure 11 shows that low $L^0$ or $N^0$ causes phytoplankton extinction since light and nutrients are two essential resources for phytoplankton growth. With the gradual increase of $L^0$ or $N^0$, phytoplankton first invade aquatic ecosystems, after which lytic viruses begin to spread among phytoplankton. When $L^0$ or $N^0$ is at a high value, the system undergoes a Hopf bifurcation and exhibits periodic oscillations. This is a classical paradox of enrichment.

The combined effects of $L^0$ and $N^0$ are presented in Fig. 12. Using the basic ecological reproductive index $R_p$ and basic reproduction number $R_0$ to distinguish dynamic behavior, we take $L^0$ and $N^0$ as the coordinate parameters to divide the first quadrant of the $L^0-N^0$ plane into four regions $\Delta _i$ for $i=1,2,3,4$. Phytoplankton go extinct in $\Delta _1$ ($R_p<1$); Phytoplankton successfully invade aquatic habitats, while lytic viruses cannot survive in $\Delta _2$ ($R_p>1$ and $R_0<1$). If $R_p>1$ and $R_0>1$, lytic viruses spread among phytoplankton as a steady state form in $\Delta _3$ or as a spatially inhomogeneous periodic solution form in $\Delta _4$. These findings suggest that it is very difficult for lytic viruses to spread in a low-light or oligotrophic aquatic ecosystem.

## Conclusion and discussion

Viruses are pervasive components of aquatic ecosystems, and have important influences on aquatic biodiversity and biogeochemical cycles (Suttle 2005; Suttle et al. 1990). As one of the common viruses, lytic viruses use phytoplankton cells as their primary hosts and are ubiquitous in aquatic ecosystems (Demory et al. 2021; Edwards and Steward 2018; Fuhrman et al. 2011; Kuhlisch et al. 2021). They replicate and reproduce inside phytoplankton cells, and eventually release virions by lysing the cells. The spread of lytic viruses causes a dramatic decrease in phytoplankton biomass and even terminates phytoplankton blooms. Few mathematical models have been formulated to explore the interactions between phytoplankton and lytic viruses and to expound the mechanisms of lytic virus transmission.

The dynamic model (2.1)–(2.3) is proposed to describe the spread of lytic virus among phytoplankton in a water column. Compared with the existing models in Béchette et al. (2013); Beretta and Kuang (1998); Demory et al. (2021); Edwards and Steward (2018); Fuhrman et al. (2011), there are two novel points in model (2.1)–(2.3). One is to consider a poorly mixed aquatic environment and take account of random movements of phytoplankton and viruses. The other is to add phytoplankton sinking movement and include light as a factor contributing to the growth of phytoplankton. Our results show that these newly added factors have a significant effect on the dynamics of phytoplankton growth and the spread of lytic viruses.

Two quantitative ecological indices: the basic ecological reproductive index $R_p$ for phytoplankton invasion and the basic reproduction number $R_0$ for virus transmission, are rigorously derived from the model. According to Theorems 3.3, 3.6, 3.11 and corresponding remarks, phytoplankton go extinct if $R_p<1$, phytoplankton successfully invade and lytic viruses are extinct if $R_p>1$ and $R_0<1$, lytic viruses spread among phytoplankton as a steady state form or a spatially inhomogeneous periodic solution form if $R_p>1$ and $R_0>1$. By using theoretical and numerical analysis of the model (2.1)–(2.3), we consider the interaction between phytoplankton blooms and lytic virus transmission and the role of ecological factors. From the numerical bifurcation diagrams (Figs. 7, 8, 9, 10, 11), one can observe that the spread of lytic viruses controls phytoplankton biomass. Time series diagrams (Figs. 7, 8b) reveal large-scale outbreaks of lytic virus infection effectively terminate phytoplankton blooms. The findings validate the observations and experimental results of *Emiliana huxleyi*-lytic virus interactions in Fuhrman (1999); Kuhlisch et al. (2021). The studies also indicate that it is very difficult for lytic viruses to spread in a low-light or oligotrophic aquatic environment (see Figs. 11, 12).

In this paper, we attempt to model the spread of lytic virus among phytoplankton, and the mechanism of lytic virus transmission and its relationship with phytoplankton blooms are explored. There are still many mathematical and biological problems worthy of further study. Mathematically, more dynamic properties of model (2.1)–(2.3) need to be rigorously investigated. For example, the uniqueness and stability of disease-free steady state $E_2$ and endemic steady state $E_3$, and the existence of spatially inhomogeneous periodic solutions. Biologically, the phytoplankton intracellular nutrient-to-carbon ratio in model (2.1)–(2.3) is assumed to be constant, but in reality, it is significantly variable (Loladze et al. 2000; Wang et al. 2007). It is desirable to include this factor in phytoplankton-virus interactions. Bacteria are a very important aquatic microorganism. They decompose organic carbon produced by phytoplankton and are infected by aquatic viruses (Fuhrman 1999; Suttle 2005; Wang et al. 2007; Yan et al. 2022). It is very natural to add bacteria into the model (2.1)–(2.3) and further explore the carbon cycle in aquatic ecosystems. In addition, the effects of zooplankton, fish (Chen et al. 2017) and toxins (Shan and Huang 2019; Nie et al. 2017) could also be considered.

## Data Availability

The datasets generated during and/or analyzed during the current study are available from the corresponding author on reasonable request.
